# Does Mixed Linker-Induced Surface Heterogeneity Impact the Accuracy of IAST Predictions in UiO-66-NH_2_?

**DOI:** 10.1021/acs.jpcc.3c04845

**Published:** 2023-10-12

**Authors:** Lukas W. Bingel, Zhenzi Yu, David S. Sholl, Krista S. Walton

**Affiliations:** † School of Chemical & Biomolecular Engineering, Georgia Institute of Technology, Atlanta, Georgia 30332, United States; ‡ Oak Ridge National Laboratory, Oak Ridge, Tennessee 37830, United States

## Abstract

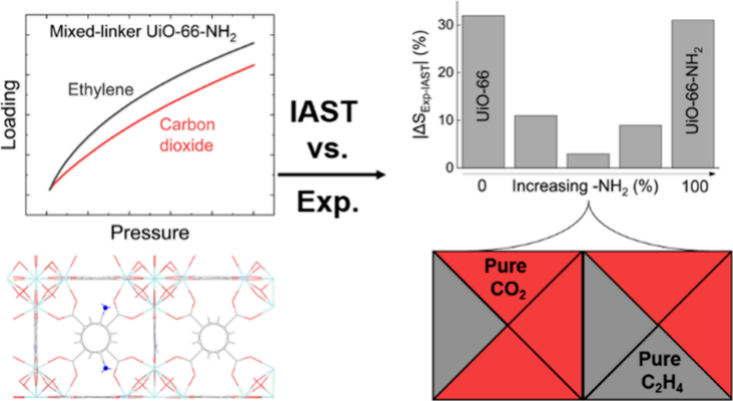

To move toward more energy-efficient adsorption-based processes, there is a need for accurate multicomponent data under realistic conditions. While the Ideal Adsorbed Solution Theory (IAST) has been established as the preferred prediction method due to its simplicity, limitations and inaccuracies for less ideal adsorption systems have been reported. Here, we use amine-functionalized derivatives of the UiO-66 structure to change the extent of homogeneity of the internal surface toward the adsorption of the two probe molecules carbon dioxide and ethylene. Although it might seem plausible that more functional groups lead to more heterogeneity and, thus, less accurate predictions by IAST, we find a mixed-linker system with increased heterogeneity in terms of added adsorption sites where IAST predictions and experimental loadings agree exceptionally well. We show that incorporating uncertainty analysis into predictions with IAST is important for assessing the accuracy of these predictions. Energetic investigations combined with Grand Canonical Monte Carlo simulations reveal almost homogeneous carbon dioxide but heterogeneous ethylene adsorption in the mixed-linker material, resulting in local, almost pure phases of the individual components.

## Introduction

By some estimates, more than 15% of the U.S. energy consumption is used for separating chemical mixtures, creating an enormous opportunity for energy savings.^1^ Unlike traditional separation methods such as distillation or drying that require significant energy or heat inputs, adsorption-based methods do not rely on bulk phase changes. Implementing adsorption-based processes in industrial settings requires reliable mixture adsorption data.^2^ Most research on adsorption-based processes tends to focus on idealized, single-component systems.^3^ However, in reality, all applications are complex mixtures by definition.^2^ A key reason for this mismatch is the complexity involved in collecting mixture adsorption data in comparison to single-component data.^4,5^

To bypass the challenge of collecting mixture adsorption data, it is common to use mixture adsorption theories, with the Ideal Adsorbed Solution Theory (IAST) as one of the most prominent examples.^6^ IAST was derived based on key assumptions: all components in the mixture have equal access to the adsorption sites, and once adsorbed, the adsorbed phase behaves like an ideal solution.^7^ However, literature reports show mixed accuracy for IAST and that not all assumptions need to be met to achieve accurate predictions.^6^ Gharagheizi and Sholl performed a comprehensive assessment of the applicability of IAST for mixture data from hundreds of experimental measurements of mixture adsorption, confirming this last point.^8^ Moreover, for more than half of the systems analyzed, the predicted selectivity values deviated up to 50% from the experimentally determined results. Wu and Sircar showed deviations of at least 40% for predicted selectivities in one-third of the 46 binary and ternary systems analyzed.^9^ Cessford et al. found that the ability of IAST to predict mixture adsorption depends on the characteristics of the adsorbent and the behavior of the adsorbate mixture.^10^ In particular, deviations can arise from an inhomogeneous distribution of adsorbate molecules within the adsorbent, which can be the result of a high degree of surface heterogeneity.^9−11^ Here, a heterogeneous surface means the distribution of different adsorption sites with different binding energies, resulting in varying binding affinities at different sites for the components of interest. This kind of heterogeneity can lead to dramatic discrepancies between IAST and mixture adsorption experiments,^12^ as observed in the case of the adsorption of carbon dioxide and water in zeolite 5A, where significant deviations were found due to surface heterogeneity arising from accessible cations.^13^ At the same time, examples are known from molecular simulations where IAST gives accurate predictions in materials that are structurally heterogeneous such as amorphous porous carbons.^14^ Despite all these comprehensive analyses, there is still a lack of systematic studies to enable a more predictive evaluation and to establish guidelines regarding the applicability and limitations of IAST.^6^

Metal–organic frameworks (MOFs) are ideal for systematic studies of adsorption in nanoporous materials due to their high tunability and modular assembly.^15^ In particular, mixed-linker MOFs, where differently functionalized linkers are combined in one structure, result in a controllable extent of surface heterogeneity imparted on the internal pore surfaces and exhibit tunable adsorption behavior.^16−18^ This mixed-linker approach has been reported for a variety of MOFs such as IRMOF-1,^19^ MIL-53,^20^ MIL-101(Cr),^21^ different ZIFs,^16,22,23^ and UiO-66.^24−26^ These multilinker materials provide a suitable platform to investigate and understand fundamental contributions to nonideal adsorption behavior and probe the limits of IAST.

For this study, UiO-66 was chosen as the baseline framework due to its stability and tunability.^17,27,28^ The framework was modified through the controlled, stepwise incorporation of amine functionalization to enable specific interactions with the selected probe molecules. Carbon dioxide and ethylene can interact with the amine groups based on their quadrupole moments and the π-double bond in ethylene.^29−31^ Moreover, similar total loadings of these probe molecules at a given pressure in UiO-66 and its derivatives suggest competitive adsorption due to similar adsorption affinities, which has been reported to result in deviations from IAST predictions, highlighting the importance of studying these systems.^10,32^ Additionally, these probe molecules are present in the off-gas mixture in acetaldehyde production, emphasizing their relevance in real applications.^33^

Using CO_2_ and ethylene as probe molecules, trends in the accuracy of IAST are investigated by comparing the loadings in binary breakthrough experiments to IAST predictions based on single-component isotherms. We show that pseudoideal local phases are formed under specific circumstances of mixture composition, total pressure, and linker ratio, resulting in good IAST predictions despite increased surface heterogeneity.

## Methods

### Chemicals and Adsorbates

Zirconium(IV)chloride, 1-4-benzenedicarboxylic acid (BDC, terephthalic acid), and 2-aminobenzene-1,4-dicarboxylic acid (BDC-amine, 2-aminoterephthalic acid) for the adsorbent synthesis as well as the solvents *N*,*N*-dimethylformamide (DMF) and methanol were procured from commercial suppliers and used without further purification (see Table S1 for suppliers and purities). All gases used for the adsorption measurements in the study (carbon dioxide, ethylene, and helium) were purchased from Airgas as well as nitrogen and air used for the characterizations described below. Purities are listed in Table S1.

### Adsorbent Synthesis and Preparation

The mixed-linker derivatives of UiO-66 with amine functionalization were synthesized following previously reported procedures changing the molar ratio of the two linkers of interest in the desired amount in the synthesis solution.^34^ Post reaction, the crystals were washed with DMF three times overnight followed by three overnight cycles of solvent exchange with methanol. The dried powders were pelletized at 10,000 psi without a binder. The resulting pellets were crushed and sieved for the 20 × 40-mesh fraction (425–850 μm) to be used for adsorption experiments.

### Material Characterization

Cryogenic nitrogen physisorption was conducted on a Quantachrome Quadrasorb SI volumetric analyzer using the pristine as-synthesized samples. The samples were outgassed at 120 °C for 24 h using a Quantachrome FloVac activation stage. BET surface area analysis was conducted using the BETSI analysis tool.^35^ Pore volumes were determined at the end of the isotherm plateau at a relative pressure of 0.90. Powder X-ray diffraction (PXRD) measurements were conducted using the powder smaller than the 20 × 40 mesh after pelletization on an X’Pert Pro PANalytical X-ray diffractometer using Cu Kα radiation (λ = 1.5418 Å) connected to an X’Celerator detector at room temperature between 4° and 50°. Reference patterns were generated using Mercury software from the Cambridge Crystallographic Data Center using a cif file of UiO-66 obtained from the CoRE MOF 2019 Database.^36^ Thermogravimetric analysis (TGA) was done on a NETZSCH STA 449 F1 Jupiter. Around 5 mg of sample was heated from room temperature to 800 °C using a ramp rate of 2 °C/min under a constant flow of nitrogen (50 mL/min) and air (20 mL/min). For ^1^H nuclear magnetic resonance spectroscopy (NMR) analysis, the samples were digested in 40 wt % NaDO in D_2_O solution overnight. After filtration, the solution was measured on a Bruker Avance III-400 NMR spectrometer. The linker ratio quantification was conducted based on the integrals of the ^1^H signals from the different linkers.

### Adsorption Experiments

Gravimetric single-component adsorption isotherms were measured on a Hiden IGA-003 Sorption Analyzer. The pelletized samples were activated *in situ* under dynamic vacuum at 120 °C for 20 h. Isotherms were collected over a range of 0 to 10 bar at 298 K for ethylene and carbon dioxide. Additional ethylene isotherms between 0 and 1 bar were measured at 288 and 308 K. Additional carbon dioxide isotherm measurements were conducted on a Micromeritics 3Flex Surface Characterization Analyzer after activation at 120 °C and under vacuum at temperatures of 288, 298, and 308 K for the heats of adsorption calculations. The experimental data were fitted using the Langmuir–Freundlich isotherm model to determine isobars that can be utilized in the Clausius–Clapeyron approach to calculate isosteric enthalpies of adsorption.^37,38^ A normally distributed error with a mean of zero and a standard deviation of 0.05 mmol/g was added to the isotherms, translating an uncertainty estimation approach used for computational calculations to the experimental results in this study.^14^ The resulting isotherms with included uncertainty were refitted to determine uncertainty intervals based on the Clausius–Clapeyron equation for the loading-dependent heats of adsorption. IAST calculations based on experimental data were conducted using IAST++ software^39^ after fitting the experimental isotherms with the dual site Langmuir–Freundlich model. The same uncertainty approximation approach described above was used for the IAST predictions. Binary mixture adsorption measurements were conducted on a home-built breakthrough instrument used in previous studies.^40^ The pelletized samples were packed in a fixed bed using glass wool and activated *in situ* at 120 °C under a constant helium flow of 5 mL/min. For the adsorption measurements, a binary mixture of ethylene and carbon dioxide was controlled by MKS Instruments Inc. mass flow controllers with a total flow rate of 10 mL/min at 298 K. The ratio was adjusted based on the desired mixture composition at 1 bar total pressure including the helium carrier flow rate. The outlet concentration was monitored with a Hiden Analytical DSMS Gas Analysis System. After subtraction of the dead volume from a blank column run using sand of the same particle size, the area above the curve is integrated to determine the dynamic capacities for the individual components.^41^

### Structure Generation

Structures for the three mixed-linker derivatives were generated by manually adding amine groups to the original BDC linkers. The original cif file was obtained from the CoRE MOF database, where RUBTAK is the associated code for UiO-66.^36^ For each mixed-linker concentration, five structures were relaxed using spatially periodic DFT in the Vienna ab initio simulation package^42^ with a plane-wave basis set and projected-augmented wave pseudopotentials.^43^ All calculations used the Perdew, Burke, and Ernzerhof generalized gradient approximation exchange-correlation functional^44^ with D3 dispersion correction (PBE-D3).^45^ All DFT calculations used a single unit cell of UiO-66 containing 24 Zr atoms as the computational volume. We simultaneously optimized both the lattice parameters and atomic positions by using a plane-wave cutoff energy of 600 eV and Γ-point sampling in reciprocal space. Using a quasi-Newton method, we relaxed geometries until the force on each atom was smaller than 0.05 eV/Å. Point charges on atoms in each of the resulting structures were assigned using the DDEC6 method.^46^ The resulting structure files used in this work are attached as cif files in the Supporting Information.

We assumed in generating mixed-linker structures, that linkers are randomly mixed within the UiO-66 structure. It is typically challenging to assess this issue quantitatively with experiments. Careful studies of mixed-linker ZIFs have demonstrated near-random distribution of linkers,^47,48^ but similar data is not available for mixed-linker UiO-66. Systematic deviations from random ordering of metal centers in heterometallic lanthanide MOFs have been suggested, but this effect stems from the different geometric binding preferences among lanthanide metals.^49,50^ To partially address this issue, we generated five different configurations for each mixed-linker ratio and used them to calculate the adsorption properties.

### Adsorption Simulation

Grand Canonical Monte Carlo (GCMC) simulations were used to simulate adsorption isotherms. These simulations used the RASPA software^51,52^ and employed the TraPPE force fields^53,54^ to describe the van der Waals (vdW) interactions between adsorbates. Adsorbate–MOF interactions were defined using Lorentz–Berthelot mixing rules, with the vdW parameters for framework atoms obtained from the UFF4MOF (for metal atoms) and DREIDING (for other atoms) (Table S2).^55−57^ The choice of the force field was validated in the previous meta-data analysis by Park et al.^58^ Lennard-Jones interactions were truncated at 12 Å, and Coulombic interactions were modeled using the long-range Ewald summation scheme with a relative accuracy of 10^–6^. MOF unit cells were replicated to a minimum of 24 Å along each dimension under triclinic periodic boundary conditions in all dimensions. The MOF structure, including functional groups on linkers, was assumed to be rigid during the GCMC simulations. It has been shown previously that this assumption of rigidity is appropriate for adsorption in unfunctionalized UiO-66.^59^ Monte Carlo trial moves, including translation, rotation, reinsertion, deletion, and insertion moves, were attempted with equal probabilities during GCMC. The simulations used 10^5^ equilibration and 10^5^ production cycles, which were shown to yield well-converged results in initial tests. For the mixed-linker MOFs, the adsorption isotherms are averaged over five different configurations generated as defined above. The simulation input files used for this work are included in the Supporting Information.

## Results and Discussion

The systematic introduction of specific amounts of amine-functionalized terephthalic acid linkers into the UiO-66 structure engenders a controllable degree of binding site heterogeneity. We synthesized five materials: two single-ligand and three mixed-linker adsorbents with 25, 50, and 75% of the amine-functionalized linker in the synthesis solution. The nomenclature used follows the structure UiO-66-NH_2_ [mol % BDC]:[mol % BDC-amine], where the mole percentages are the concentrations in the synthesis solution.

**Figure 1:**
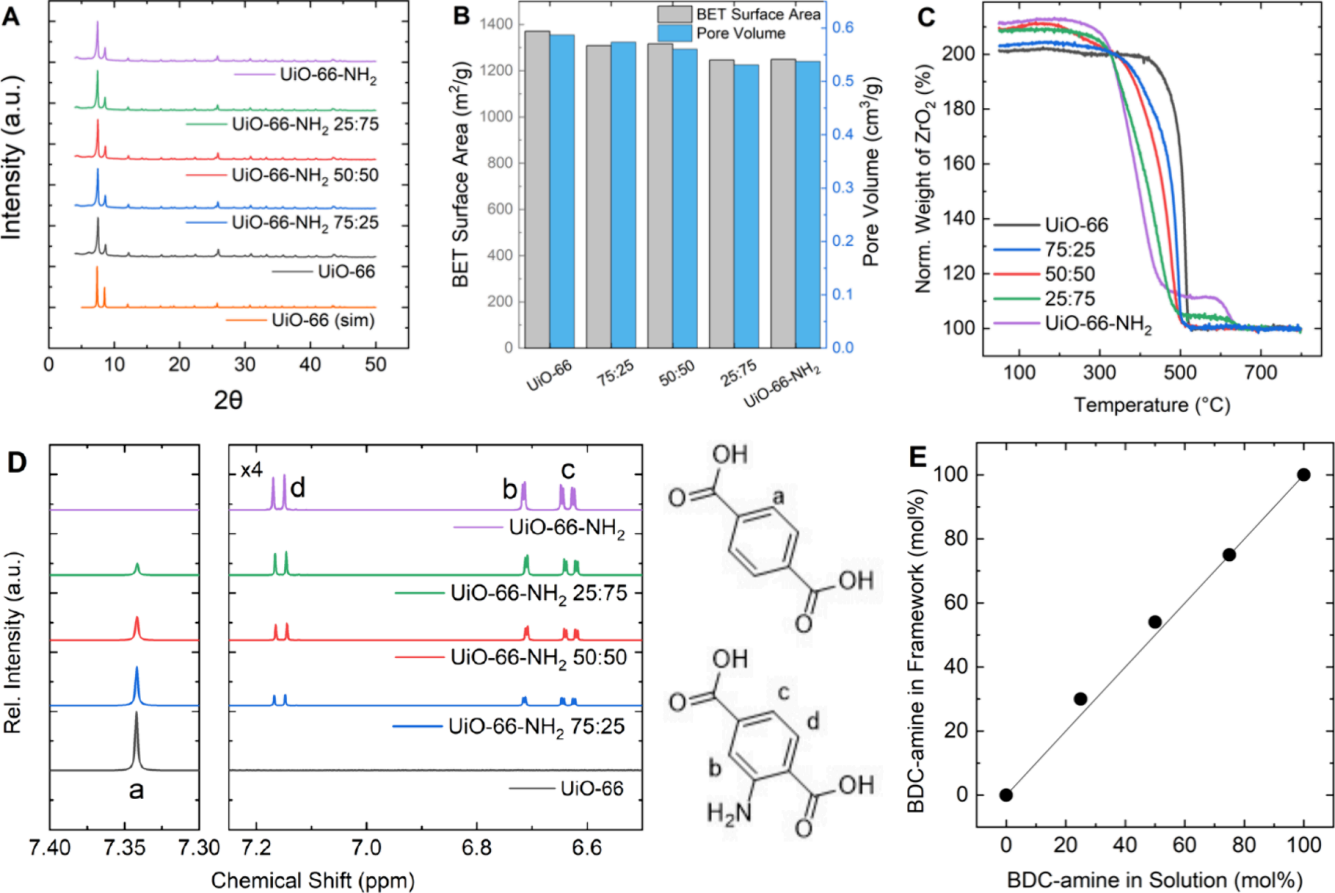
Characterization of the mixed-linker UiO-66-NH_2_ derivatives. (A) Powder X-ray diffraction pattern. (B) BET surface areas and pore volumes determined from cryogenic nitrogen physisorption. (C) Thermogravimetric analysis up to 800 °C. (D) Digestion proton NMR results, with the right half magnified by a factor of 4 for clarity. Protons on the two ligands labeled according to the assignment in the NMR spectra. (E) Quantitative analysis of the NMR spectra and comparison of amount of amine-functionalized ligand in the synthesis solution and incorporated in the MOF structure.

As seen in Figure 1A, the powder X-ray diffraction patterns for all samples show good agreement with the pattern simulated for unfunctionalized UiO-66. No changes of the characteristic fingerprint of the patterns due to the functionalization are observed, as expected for a series of isoreticular structures. The characteristic reflection peaks slightly shift to lower 2θ as more amine groups are added (Figure S1), although this outcome was not seen in our DFT calculations. The experimental data suggests that the bulky functional groups enlarge the unit cell and, thus, the lattice, resulting in changes in the PXRD patterns.^26^ The shift is linearly correlated to the amount of BDC-amine in the structure (Figure S1), consistent with our assumption of a homogeneous distribution of the different ligands on the unit cell scale.^60^

BET surface areas and pore volumes in Figure 1B were determined from cryogenic nitrogen physisorption measurements (Figure S2) and analyzed using the BETSI tool^35^ (Figures S3–S7). Both properties decrease with increased functionalization due to additional amine groups taking up void space.^17,61^ The BET surface area of 1370 m^2^/g for unfunctionalized UiO-66 is higher than the value simulated in an ideal, defect-free crystal structure of 1093 m^2^/g but within commonly reported ranges.^62^ The higher surface area in the experimental determination can be attributed to defects for which the UiO-66 framework is known.^63^

Since the existence of defects influences adsorption behavior,^64^ thermogravimetric analysis was conducted to quantify the missing linker defects in the each material. After an initial loss of adsorbed water and solvent molecules below 100 °C, the TGA curves for all five materials show a complete lattice breakdown and full linker decomposition up to 500 °C, as shown in Figure 1C. A stepwise decrease in the decomposition temperature can be observed as the amount of the amine-functionalized terephthalic acid linker increases.^28,61^ The materials with large amounts of incorporated amine groups show an additional step around 600 °C from the decomposition of a zirconium nitride component that formed as an intermediate product during the heating process. The magnitude of this step qualitatively relates to the amount of −NH_2_ groups in the framework. While it is obviously visible for the fully functionalized UiO-66-NH_2_, the step is much smaller for the 25:75 derivative and is absent for the less functionalized materials. The curves for all materials plateau above 650 °C after a complete oxidation to ZrO_2_.^64^ Following an approach of Shearer and co-workers,^64^ we used TGA curves to quantify the missing linker defects, finding 1.0–1.1 missing linker per Zr_6_ unit for all five materials (Table S3). This observation means that differences in adsorption between the materials can be attributed to changes due to the presence of amine groups rather than a changed extent of defects in the structures.

To quantify the amounts of the two terephthalic acid derivatives in the mixed-linker frameworks, digestion ^1^H NMR was conducted. Figure 1D visually shows the trends of the peak labeled as “a” corresponding to the four protons with the same chemical environment in the BDC linker decreasing and ultimately disappearing as more functionalized linkers are introduced and finally forming the fully functionalized UiO-66-NH_2_ structure. The opposite is shown for the protons “b”, “c”, and “d” from the BDC-amine linker, which increase as more of the linker is incorporated in the structures. The quantitative analysis in Figure 1E shows slightly higher amounts of the functionalized linker in the structure than used in the synthesis solution, but these deviations are small enough that labeling the structures according to the composition of the synthesis solution is reasonable.

**Figure 2:**
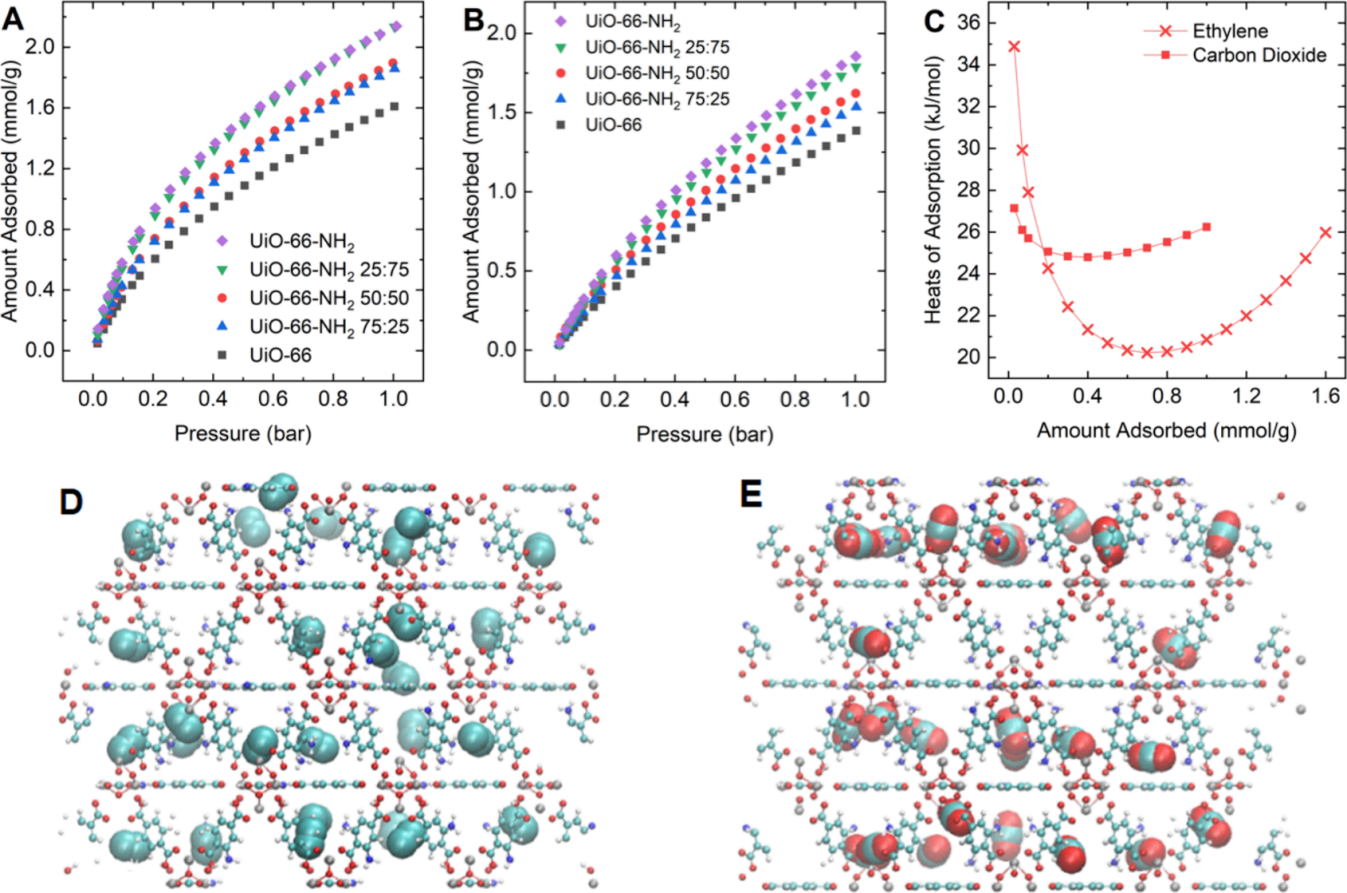
Single-component adsorption of ethylene and carbon dioxide. Single-component isotherms at 298 K in the five materials for (A) ethylene and (B) carbon dioxide. (C) Loading-dependent isosteric heats of adsorption for both adsorbates in the UiO-66-NH_2_ 50:50 mixed-ligand derivative determined from isotherms measured at 288, 298, and 308 K. Visualization of 0.5 mmol/g adsorbed of (D) ethylene and (E) carbon dioxide at 298 K from GCMC simulations in the UiO-66-NH_2_ 50:50 mixed-linker material. Oxygen atoms are shown in red, carbon in turquoise, and nitrogen in dark blue. Hydrogen atoms have been omitted for better visibility. Adsorbate molecules are magnified to stand out against the framework atoms.

Single-component isotherms for carbon dioxide and ethylene were collected at 298 K from 0 to 10 bar gravimetrically. Figure 2A,B shows these isothermal loading curves with pressures of 0–1 bar (full isotherms in Figure S8) due to the focus of this study on low and moderate pore loadings rather than pore filling. The isotherm for UiO-66 agrees well with reported consensus isotherms from the literature meta-analysis for this system (Figure S9). The order of the isotherms changes toward higher pressures since the additional amine groups occupy void space, reducing the total capacity (Figure S8).^26^ For the pressure range shown in Figure 2A,B, the adsorbed amounts for both components increase going from the pristine UiO-66 with the lowest loading to the fully functionalized UiO-66-NH_2_ as more amine groups provide favorable adsorption sites.^17,30^ For the ethylene isotherms, the similarity of the pair of 75:25 and 50:50 isotherms and the pair of 25:75 and UiO-66-NH_2_ isotherms can be attributed to the opposing effects of increased adsorption affinity from the amine groups and their steric hindrance to the bulkier ethylene molecules.^32^ The isotherms for both adsorbates in all five adsorbents show a type I behavior indicative of the presence of strong adsorbate–adsorbent interactions with specific adsorption sites on the internal surface.^65^ The isotherms for ethylene increase more rapidly with pressure compared to carbon dioxide in the low pressure range, suggesting stronger interactions with the highest energy adsorption sites.

To quantify these interactions, additional isotherms were measured at 288 and 308 K in the two pure MOFs as well as in the 50:50 mixed-linker MOF (Figure S10). The Clausius–Clapeyron approach was utilized to determine loading-dependent heats of adsorption in the three materials for both adsorbates, shown for the UiO-66-NH_2_ 50:50 derivative in Figure 2C. Fitting parameters for the Langmuir–Freundlich isotherm used to model the experimental data are tabulated in Tables S4 and S5. The mixed-ligand surface is significantly energetically heterogeneous for ethylene adsorption, as seen from the concave curve compared to both pristine MOFs (Figure S11) where we find continuously decreasing heats of adsorption with increasing loadings over a smaller amplitude compared to the mixed-ligand adsorbent. At near-zero loadings, ethylene molecules adsorb with energies of up to 35 kJ/mol on the metal nodes, which is comparable for all three materials considering the uncertainty intervals for low loadings.^31^ The heats of adsorption decrease to ∼20 kJ/mol at an ethylene loading of about 0.7–0.8 mmol/g and then increase again due to intermolecular interactions between adsorbed molecules via π–π interactions. The heats of adsorption for ethylene are higher than that for carbon dioxide for loadings smaller than ∼0.2 mmol/g, but the reverse is true for higher loadings.

The heats of adsorption for carbon dioxide only vary between 25 and 27 kJ/mol, revealing a relatively homogeneous energy landscape for carbon dioxide adsorption in the mixed-ligand material. The carbon dioxide heats of adsorption at almost zero loading are similar in all materials within experimental uncertainties (see Figure S11). For higher loadings, the heats of adsorption in the pure materials are slightly more heterogeneous compared to that of the mixed-linker derivative. The heats of adsorption for carbon dioxide result from three competing effects: introduction of high-affinity sites with increased energetic interactions to the amine groups, steric hindrance from the amine groups accessing the sites on the metal nodes, and an adsorption-promoting confinement effect with pore size reduction due to addition of amine groups.^17,66,67^ The heats of adsorption for carbon dioxide decrease to a minimum at around 0.3–0.4 mmol/g, which roughly corresponds to an occupation of all metal nodes and amine groups with one carbon dioxide molecule. The subsequent increase in heats of adsorption at higher loadings can be attributed to stronger intermolecular interactions between adjacent CO_2_ molecules in the adsorbed phase.

GCMC simulations were conducted to further understand adsorption in these materials. The use of generic force fields has been shown to lead to quantitative deviations between experimental consensus isotherms and simulated isotherms, especially for polar molecules.^58,68^ Elimination of quantitative discrepancies can be reached by fine-tuning the simulation parameters, which is beyond the scope of this study. Thus, we focused on visualizing the adsorption configuration at specific loadings of the adsorbing components. Additional information about the exploration of the pressure space using GCMC can be found in the Supporting Information. The spatial distribution of 0.5 mmol/g of ethylene and carbon dioxide in single-component adsorption in the 50:50 mixed-linker adsorbent is presented by representative snapshots in Figure 2D,E, respectively. This loading corresponds roughly to the minimum in heats of adsorption for both components in our experimental data. Ethylene molecules adsorb individually and are distributed among specific sites, i.e., the zirconium metal nodes^31^ and the amine groups on the functionalized linkers (as seen in the radial distribution functions in Figure S12), aligning with the observation of the concave heats of adsorption in Figure 2C. Ethylene adsorption in the pure MOFs occurs in clusters (Figure S13), agreeing with the more homogeneous heats of adsorption curves in these materials (Figure S11). This clustering mechanism can also be observed for carbon dioxide in the 50:50 mixed-linker framework as shown in Figure 2E. The first carbon dioxide molecules adsorb to the metal nodes.^69^ Subsequent molecules do not occupy all metal nodes but bridge from the initially adsorbed molecules on the metal nodes to the adjacent amine groups.^70^ The stronger intermolecular interactions from their higher quadrupole moment compared to ethylene facilitate this clustering.^32^ The absence of amine groups on all linkers reduces the steric limitation to form these clusters around the metal node in the fully functionalized amine-UiO-66, resulting in a more heterogeneous carbon dioxide adsorption in this material (Figures S11 and S13).

**Figure 3:**
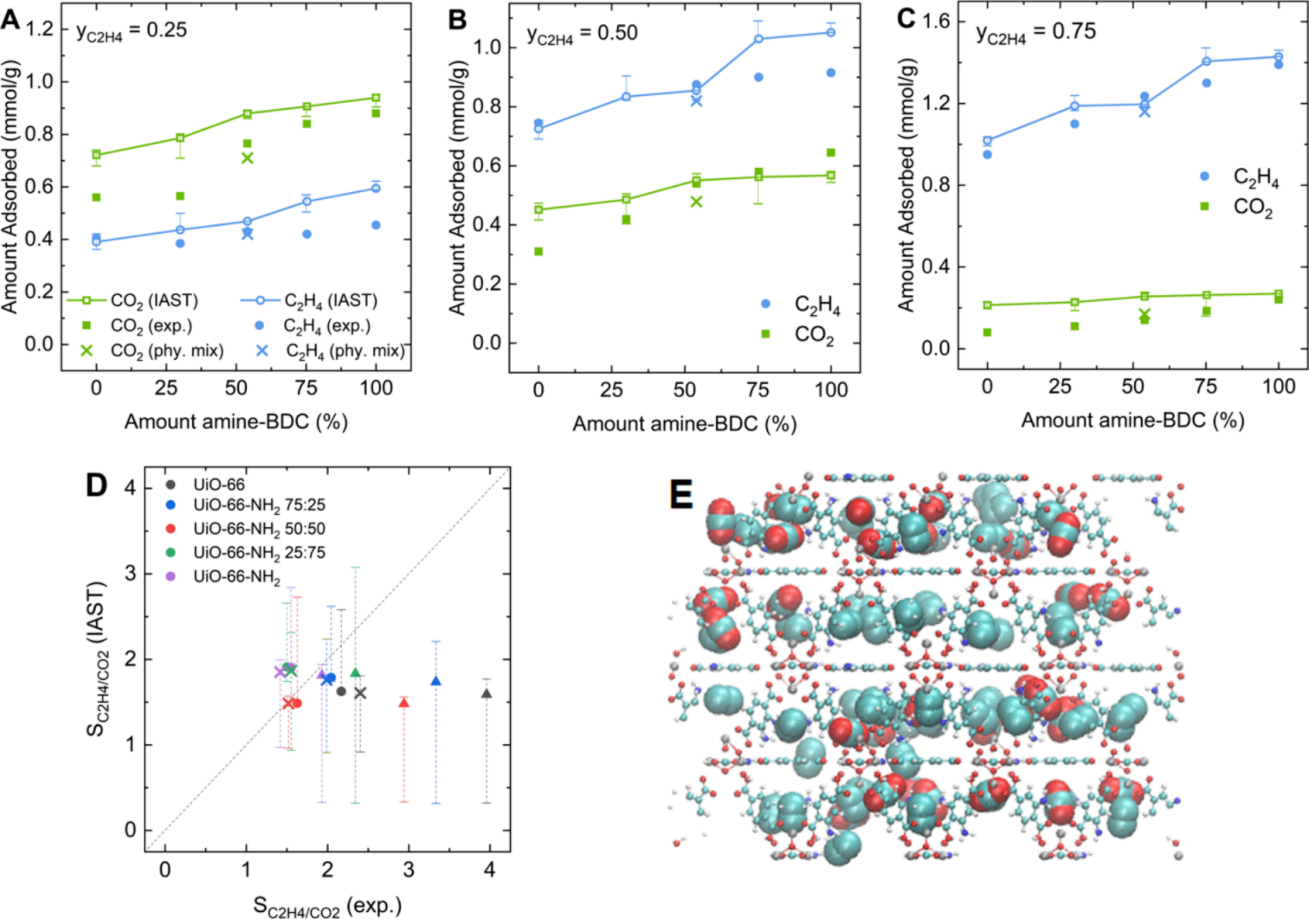
Binary coadsorption in the five UiO-66-NH_2_ derivatives. Comparison of IAST predictions to experimental results from dynamic breakthrough experiments with different gas phase compositions: (A) *y*_ethylene_ = 0.25, (B) *y*_ethylene_ = 0.50, and (C) *y*_ethylene_ = 0.75. Solid circles show breakthrough results, and open symbols show IAST predictions connected with lines to guide the eye. Error bars show minima and maxima for repeating IAST predictions with simulated uncertainty added to experimental isotherm data. Cross symbols are results from dynamic breakthrough results in physical mixtures of the two pure MOFs. (D) Comparison of IAST-predicted and experimentally determined selectivities at 0.667 bar total pressure of ethylene over carbon dioxide in the five materials, with symbols corresponding to different gas phase compositions: solid up-pointing triangle, *y*_CO2_ = 0.25; cross mark, *y*_CO2_ = 0.50; solid circle, *y*_CO2_ = 0.75. Error bars show minima and maxima calculated from error bars in IAST predictions for the individual components. (E) Visualization of GCMC simulations of the loadings in the 50:50 mixed-linker adsorbent for an equimolar mixture at 0.667 bar of total pressure. Oxygen atoms are shown in red, carbon in turquoise, and nitrogen in dark blue. Hydrogen atoms are omitted for better visibility. Adsorbate molecules are magnified to stand out against the framework atoms.

The accurate pressure and composition control possible with a dynamic column make this approach a good choice for systematically obtaining mixture adsorption data.^5^ Validation of the breakthrough approach was conducted, measuring single-component breakthrough curves and comparing the capacities to the static isotherm measurements (Figure S14). Mixture data from breakthrough experiments was compared with IAST predictions made using our experimental single component isotherms. Uncertainties for IAST predictions were estimated by ascribing a randomly sampled uncertainty with a mean of zero and a standard deviation of 0.05 mmol/g and repeating the IAST calculations with the fits of the data including uncertainties. IAST qualitatively predicts the experimentally observed trends in loadings for each species (Figure 3A–C). Predictions including uncertainties agree with measured capacities for some individual points, but a general overestimation for both components can be observed. The IAST predictions for ethylene loadings are in all cases ±25% of the experimental data. For carbon dioxide loadings, however, overestimations by IAST of up to 160% are found in some cases with low carbon dioxide loadings. Even in carbon dioxide-rich environments, overpredictions by IAST of more than 25% for the carbon dioxide loading are found in the unfunctionalized and 75:25 mixed-linker material. Additional breakthrough measurements in physical mixtures of the two pure MOFs following the composition determined using NMR spectroscopy show differences when compared to the mixed-linker materials, as shown in Figure 3A–C, suggesting synergy effects in the mixed-linker materials.

The accuracy of IAST predictions for carbon dioxide loadings generally increases with the degree of amine-functionalization and is, overall, the highest for equimolar mixtures. The only case of underestimation of the carbon dioxide loading using IAST including uncertainties compared to experiments was for an equimolar mixture adsorbing in UiO-66-NH_2_. For ethylene, the IAST accuracy is higher with less functionalization of the MOF, especially in ethylene-poor mixtures. Under these conditions, ethylene loadings are significantly overpredicted by IAST in materials with a high degree of functionalization. Agreement within uncertainties for both components is only found in the first case, namely, the 50:50 mixed-linker material exposed to an equimolar gas phase.

The deviations between IAST predictions and experimental data are more pronounced upon examination of the adsorption selectivities for each case we considered (see Figure 3D). Although the uncertainties in the IAST predictions are quite large due to the range between the minima and maxima of the dynamic capacities of the individual components, it is clear that IAST does not capture the experimentally observed variations in selectivity as a function of the gas phase composition. For equimolar mixtures, we find agreement between IAST and experimental selectivities, within prediction uncertainties, in all materials except for the unfunctionalized UiO-66. Significant underestimation of selectivity using IAST was found under ethylene-rich conditions (▲ in Figure 3D) in materials with no or low functionalization. In these cases, the uncertainty includes selectivity values <1, meaning that IAST cannot unambiguously predict which of the two components is selectively adsorbed once uncertainty is considered. Under carbon dioxide-rich conditions (● in Figure 3D), selectivity values for materials with amine amounts from 0 to 50% agree with experimental results within the given uncertainty range, but an overestimation is found for high degrees of functionalization.

Coadsorption of ethylene and carbon dioxide at loadings equivalent to those observed with equal gas phase fractions from the breakthrough experiments (Figure 3B) was simulated for the two pure MOFs as well as the 50:50 mixed-linker derivative and visualized. Figure 3E shows the coadsorption values of 0.54 mmol/g of carbon dioxide and 0.87 mmol/g of ethylene in the mixed-linker material. Compared to the pure MOFs that show more homogeneously mixed adsorbed phases throughout the entire framework (Figure S15), we find locally rather uniform and pure phases of one individual component within the pore cavities and around the adsorption sites. The Supporting Video of several cycles of Monte Carlo moves shows even more visually the random placement of carbon dioxide molecules compared to the more static ethylene phases that adsorbed iteratively at the same adsorption sites. This qualitative analysis supports the idea that the IAST assumption of an ideal adsorbed phase is met locally without competitive effects, even though the mixed-linker nature of the MOF may create local structural inhomogeneities.

## Conclusions

The Ideal Adsorbed Solution Theory is one of the best methods for determining much needed mixture adsorption data from pure adsorption data to investigate the suitability of adsorption-based processes under industrial settings. However, there are numerous examples in which the applicability of IAST is limited, which poses the need for systematic studies to investigate the limitations and accuracy. The assumptions underlying IAST suggest that using IAST may be the most appropriate when the adsorption environment is homogeneous. In this paper, we explored this idea by using mixed-linker derivatives of UiO-66 with amine-functionalized linkers that introduce surface heterogeneities (via chemical functionality) in a controlled way. We performed single-component and mixture adsorption experiments for ethylene and carbon dioxide in a range of these MOF materials.

If an increase in pore heterogeneity led to IAST being less accurate, the application of IAST to UiO-66 with 50% of the MOF’s linkers functionalized with an amine would be expected to be less accurate than that for an unfunctionalized or completely functionalized MOF. Interestingly, our results show the opposite outcome, with IAST giving the most reliable outcomes for ethylene/carbon dioxide in partially functionalized materials. These results indicate that synergy and mixture adsorption effects can compensate for the reduction in surface homogeneity.

Our analysis included an estimation of the uncertainty in IAST predictions associated with uncertainties in the measured single-component isotherms. The importance of these effects is especially significant when the adsorbed loading of one species of interest is low. In a small number of cases, IAST could not unambiguously predict which species would be preferentially adsorbed once uncertainty was included, even though our mixture experiments showed clear selectivity for ethylene relative to that for carbon dioxide.

Our results highlight the value of developing guidelines for the applicability for IAST by conducting systematic studies or comprehensive analyses of available databases. Previous work and this study have shown that underlying assumptions do not need to be met for accurate predictions. To the contrary, this work suggests that IAST may be applicable to a broader range of systems where inaccuracies were expected. Guidelines from controlled assessment of the intricacy of multicomponent adsorption system will be helpful in identifying these cases. This is especially relevant when high precision predictions are needed for process design, and accurate estimates of selectivities are required. Our work also points to the need to further expand the understanding of mixture adsorption from a fundamental standpoint on the molecular level. Here, the improvement and optimization of simulation and prediction tools are as important as the experimental investigation under more and more realistic conditions.^2^

## Supporting Information Available

Method for defect quantification, discussion of GCMC simulations, additional ethylene and carbon dioxide isotherms, heat of adsorption curves in pure MOFs, tabulated isotherm values, and tabulated isotherm fitting parameters (PDF)
Mixed-linker UiO-66-NH_2_ structures (ZIP)
Simulation inputs (ZIP)
GIFs of cycles of Monte Carlo moves for GCMC simulations (ZIP)
