# Supplementary material for: Does Mixed Linker-Induced Surface Heterogeneity Impact the Accuracy of IAST Predictions in UiO-66-NH2?
Source: J Phys Chem C Nanomater Interfaces. 2023 Oct 12;127(42):20881–9. doi: 10.1021/acs.jpcc.3c04845 (PMC10614300; doi:10.1021/acs.jpcc.3c04845)
Supplement: Supplementary file 1 — jp3c04845_si_001.pdf [file jp3c04845_si_001.pdf]

Supporting Information for

Does Mixed-Linker Induced Surface Heterogeneity Impact the Accuracy of IAST Predictions in UiO-66-NH_2?

*Lukas W. Bingel[1], Zhenzi Yu[1], David S. Sholl[1,2], Krista S. Walton[1]**

[1]School of Chemical & Biomolecular Engineering

Georgia Institute of Technology, Atlanta, GA 30332, United States

[2]Oak Ridge National Laboratory, Oak Ridge, TN 37830, United States

Corresponding Author

*E-Mail: krista.walton@chbe.gatech.edu

KEYWORDS: adsorption, metal-organic frameworks, IAST

Table of Contents

**S1**	**Supplementary Methods**		S3
S1.1	Defect quantification based on thermogravimetric analysis		S3
**S2**	**Supplementary Discussion**		S4
S2.1	GCMC Simulations		S4
**S3**	**Supplementary Figures**		S5
	Figure S1	PXRD of main peak around 2θ = 7.5	S5
	Figure S2	Nitrogen isotherm at 77 K	S5
	Figure S3	BETSI Analysis for UiO-66	S6
	Figure S4	BETSI Analysis for UiO-66-NH_2 75:25	S7
	Figure S5	BETSI Analysis for UiO-66-NH_2 50:50	S8
	Figure S6	BETSI Analysis for UiO-66-NH_2 25:75	S9
	Figure S7	BETSI Analysis for UiO-66-NH_2	S10
	Figure S8	Isotherms up to 10 bar	S11
	Figure S9	Comparison of CO_2 isotherm to consensus isotherm	S11
	Figure S10	Isotherms at 288, 298, and 308 K	S12
	Figure S11	Isosteric heats of adsorption	S13
	Figure S12	Radial distribution function of ethylene in UiO-66-NH_2 50:50	S13
	Figure S13	Visualization of GCMC simulated unary adsorption	S14
	Figure S14	Comparison of isotherms to single-component breakthroughs	S14
	Figure S15	Visualization of GCMC simulated binary adsorption	S15
	Figure S16	Thermogravimetric analysis	S15
	Figure S17	GCMC single-component isotherms	S16
	Figure S18	Comparison of experimental and simulated isotherms	S16
**S4**	**Supplementary Tables**		S17
	Table S1	Suppliers and purities of chemicals and gases	S17
	Table S2	GCMC Lennard-Jones parameters	S17
	Table S3	TGA-based defect quantification	S18
	Table S4	Fitting parameters for carbon dioxide adsorption	S18
	Table S5	Fitting parameters for ethylene adsorption	S19
	Table S6-10	Carbon dioxide isotherms at 298 K	S20
	Table S11-15	Ethylene isotherms at 298 K	S22
	Table S16	Fitting parameters for isotherms used for IAST calculations	S25
	Table S17-22	Ethylene isotherms at 288 and 308 K	S26
	Table S23-31	Volumetric carbon dioxide isotherms at 288, 298, and 308 K	S28
**S5**	**Supplementary References**		S33

S1 Supplementary Methods

S.1.1 Defect quantification based on thermogravimetric analysis

The procedure to quantify the amount of defects in the five UiO-66 derivatives used in this study follows an approach introduced by Shearer et al. for the defect generation in UiO-66 suing modulators.[1] It is based on the following general reaction for the complete combustion of dehydroxylated mixed-linker UiO-66-NH_2 under an oxidizing atmosphere. The coefficient *y* is the fraction of BDC-amine as determined from ^{1}H NMR experiments.

$$Zr_6O_6(BDC)_{6-6y}(BDC\text{-amine})_{6y}\ (s) + (45+1.5y)\ O_2\ (g) \rightarrow$$
$$6\ ZrO_2\ (s) + 48\ CO_2\ (g) + (13+3)\ H_2O + 3y\ N_2\ (g)$$

Here, the molar mass of each of the mixed-linker materials can be determined as

$$M = M\ (Zr_6O_6) + (6\text{-}6y)\ M(BDC) + 6y\ M(BDC\text{-amine}) =$$
$$[643.3 + (6\text{-}6y) \cdot 164.1 + 6y \cdot 179.1]\ g{\cdot}mol^{-1}$$

Since the starting temperature of the lattice collapse decreases with increasing amounts of BDC-amine linkers, the corresponding weight decrease starts overlapping with the weight loss attributed to solvent and water desorption (Figure S16). Thus, a constant temperature to determine the start weight $W_{ex,Pl}$ of 300 °C was selected based on the results from Shearer et al.[1] since no capping agent was used.[2]

The equations derived by Shearer et al. were used to determine the theoretical weight of the plateu $W_{theo,Pl}$, the theoretical weight contribution of each linker $W_{t,Pl,theo}$, and the experimentally-determined number of missing linkers *X* per Zr_6 unit. All these values are tabulated in Table S3.

S2 Supplementary Discussion

S2.1 GCMC Simulations

The pressure space for both adsorbates of interest, carbon dioxide and ethylene, was explored using GCMC simulations in all five generated UiO-66 derivative structures to obtain single-component isotherms at 298 K (Figure S17). A comparison of these computed isotherms to experimentally measured isotherms is shown in Figure S18. The isotherms for both adsorbates show qualitative agreement between simulations and experiments in terms of isotherm type, shape, and saturation loadings. Quantitative discrepancies can be observed since the GCMC simulated isotherms seem to be compressed along the pressure axis. These differences can be attributed to force-field differences during the simulations and differences in the defective synthesized structures compared to the defect-free, ideal simulation structure. Similar quantitative deviations have been reported before between experimental consensus isotherms from literature meta-analysis and simulated isotherms, especially for the adsorption of polar molecules.[3-4] This work does not aim to optimize the simulation approach and structures to find a quantitative match between experiments and simulations. It rather uses the simulations to generate a visualization to illustrate and understand the underlying adsorption mechanism. Thus, specific loadings of interest concluded from experiments were simulated and visualized using the GCMC simulations.

S3 Supplementary Figures

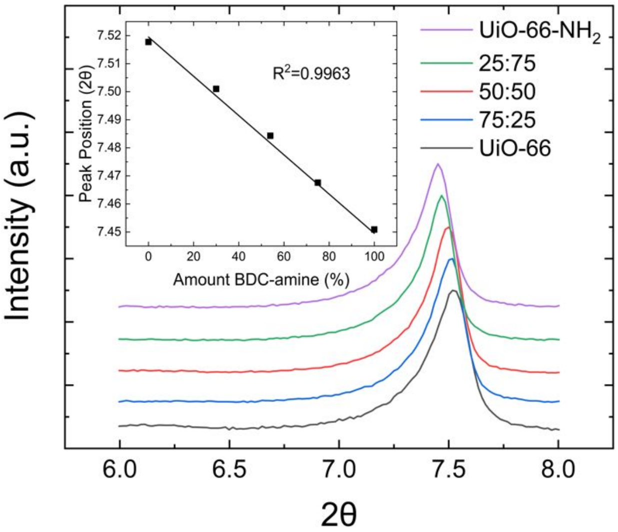

Figure S1. PXRD pattern for the main peak around a 2θ of 7.5 for all five materials. Inset shows the linear relationship between the peak position and the amount of BDC-amine linker in the structure as determine by ^{1}H NMR with the coefficient of determination for the linear relationship (peak position = 7.5195 – 0.0007 Amount BDC-amine[%])

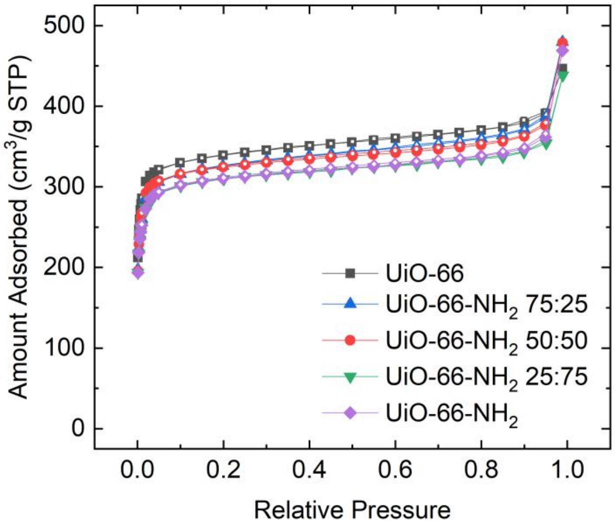

Figure S2. Nitrogen isotherms at 77 K in UiO-66, UiO-66-NH$_2$, and the three mixed-linker amine-functionalized UiO-66 derivatives.

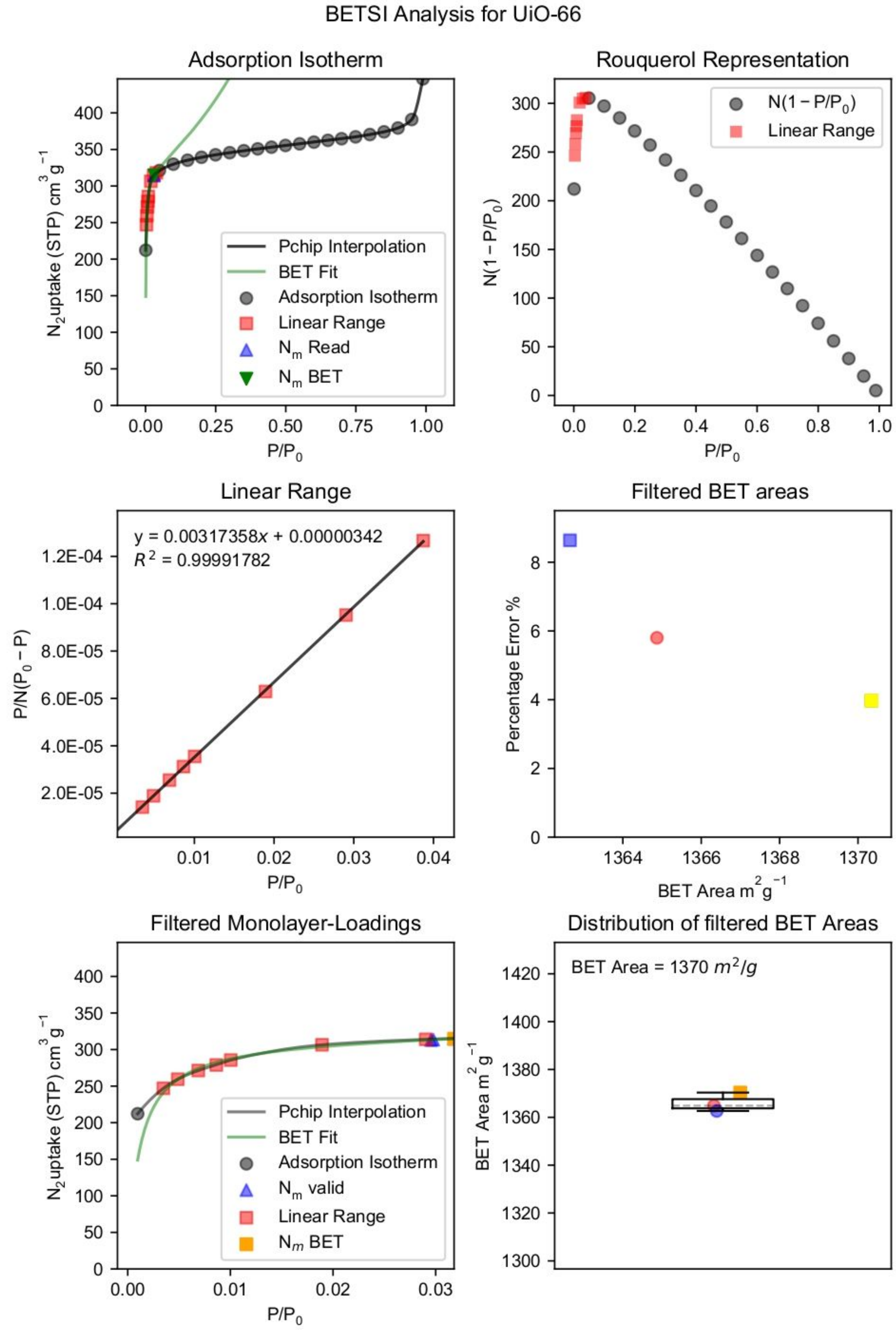

Figure S3. Results of BETSI analysis of UiO-66

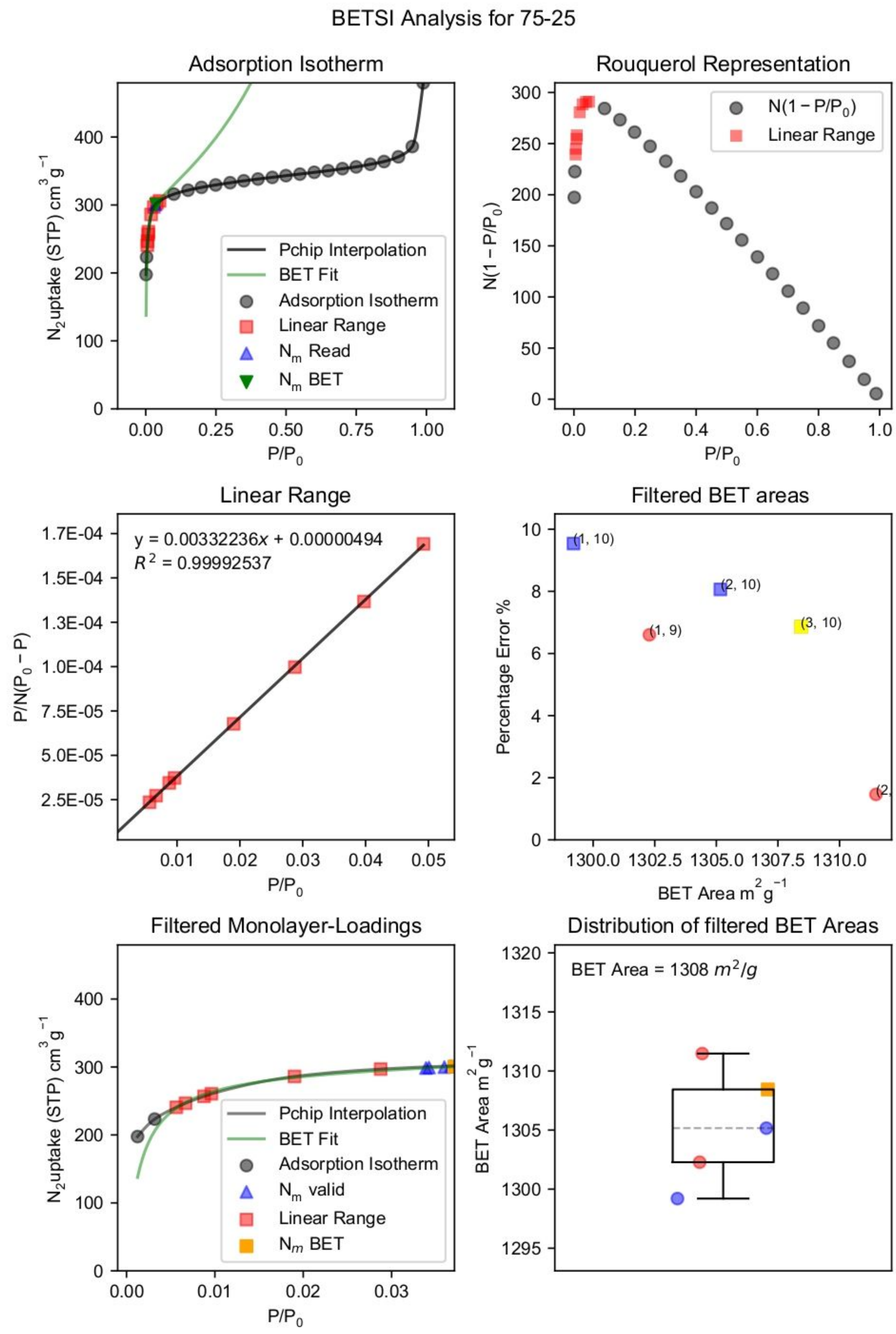

Figure S4. Results of BETSI analysis of UiO-66-NH_2 75:25

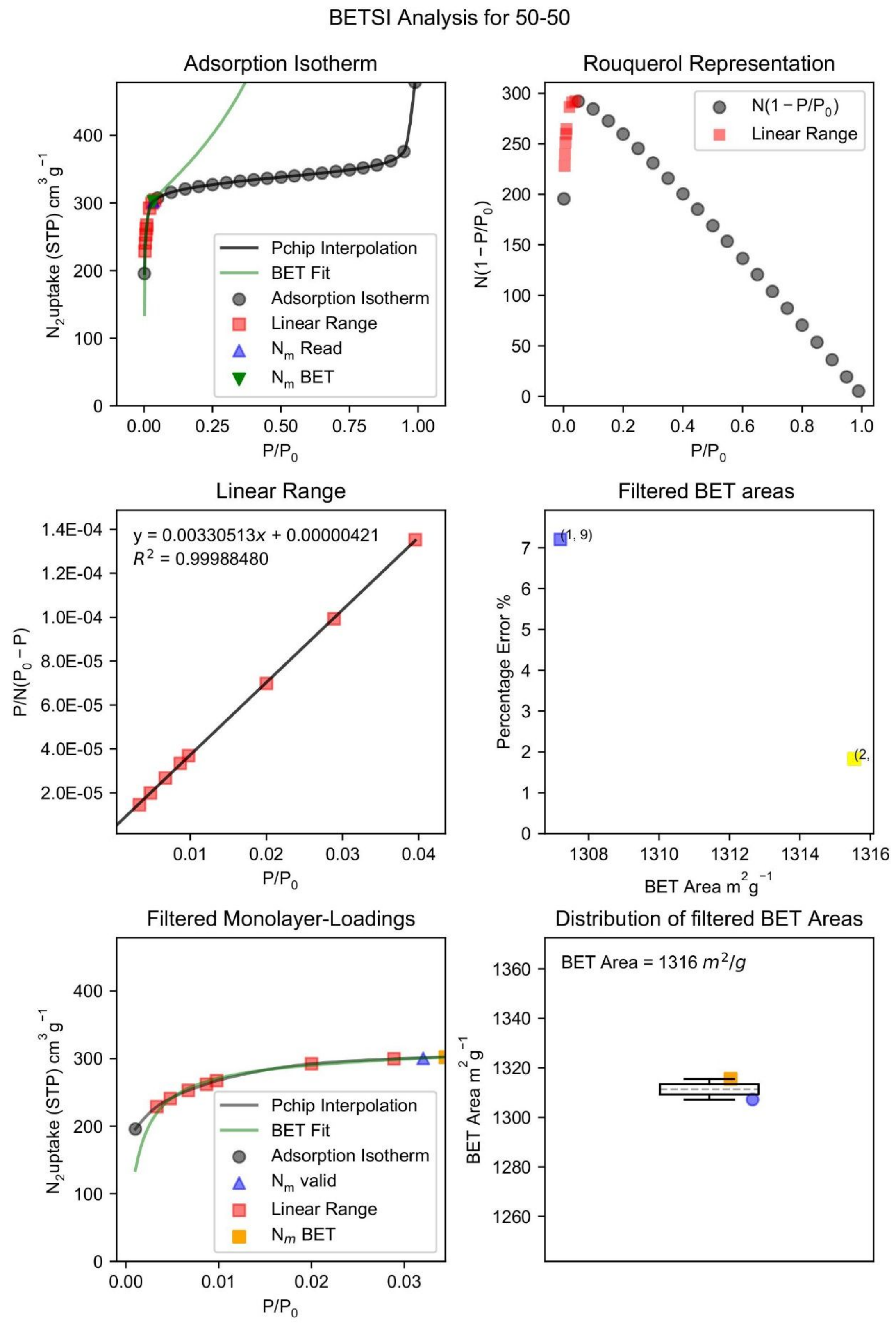

Figure S5. Results of BETSI analysis of UiO-66-NH_2 50:50

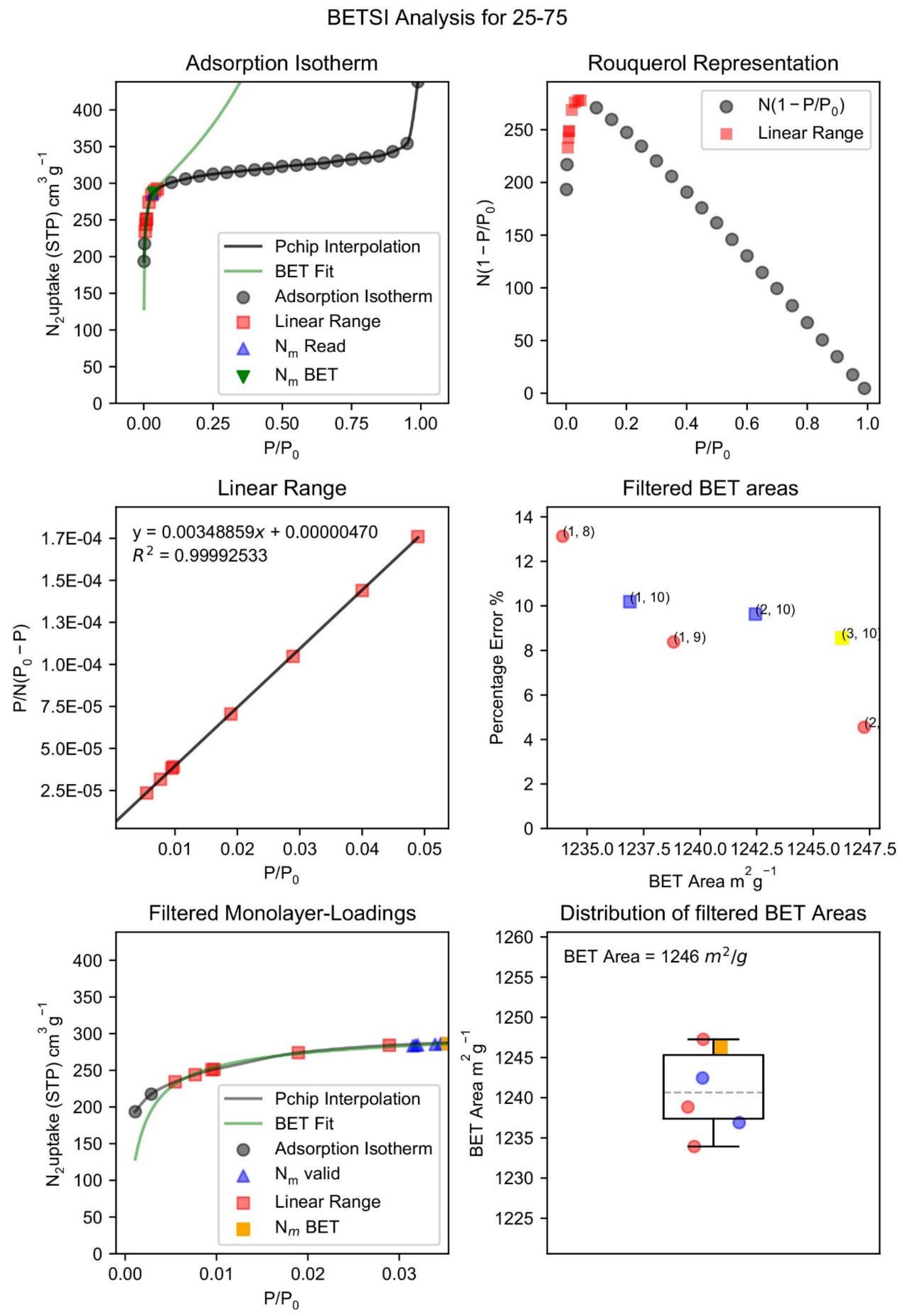

Figure S6. Results of BETSI analysis of UiO-66-NH_2 25:75

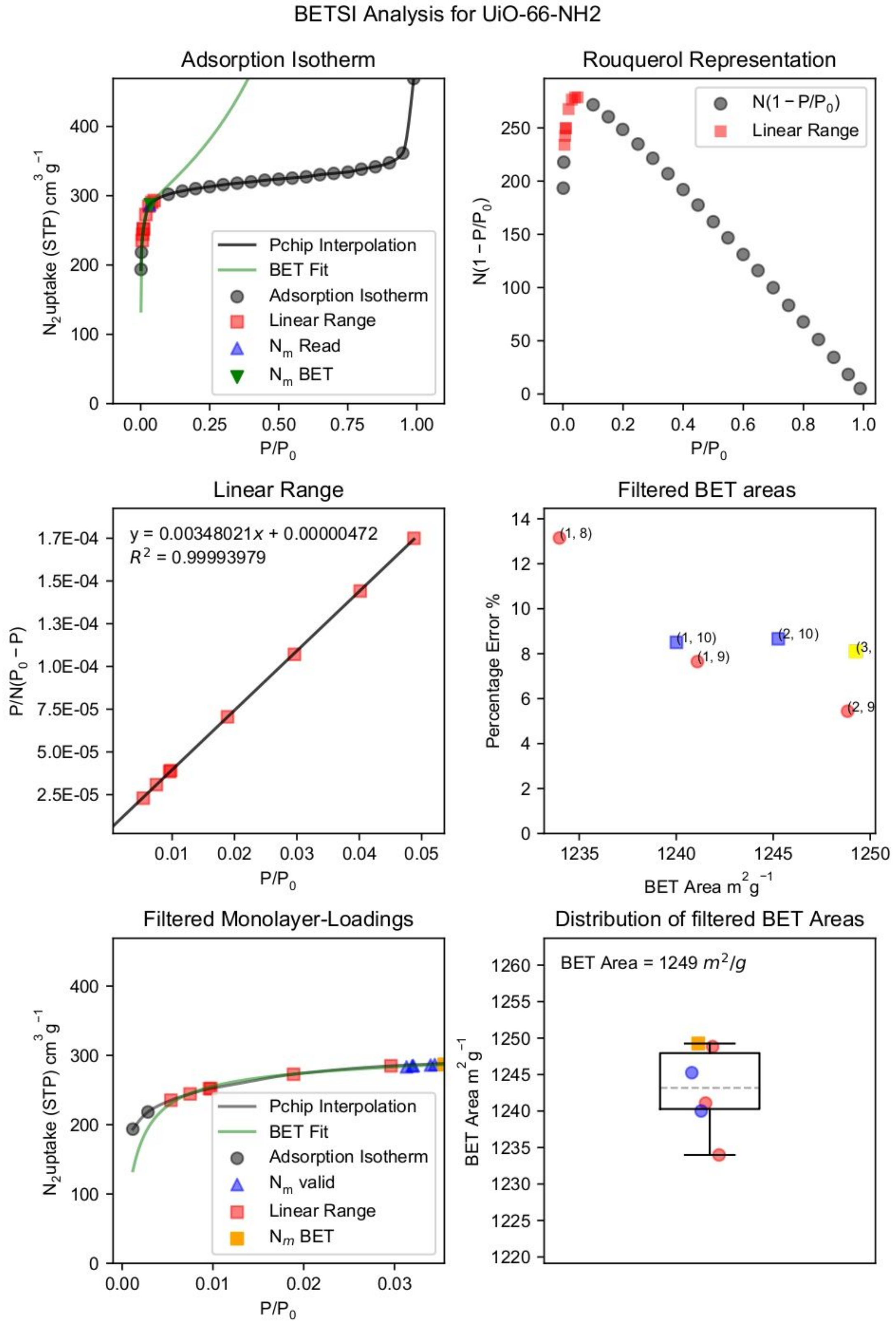

Figure S7. Results of BETSI analysis of UiO-66-NH_2

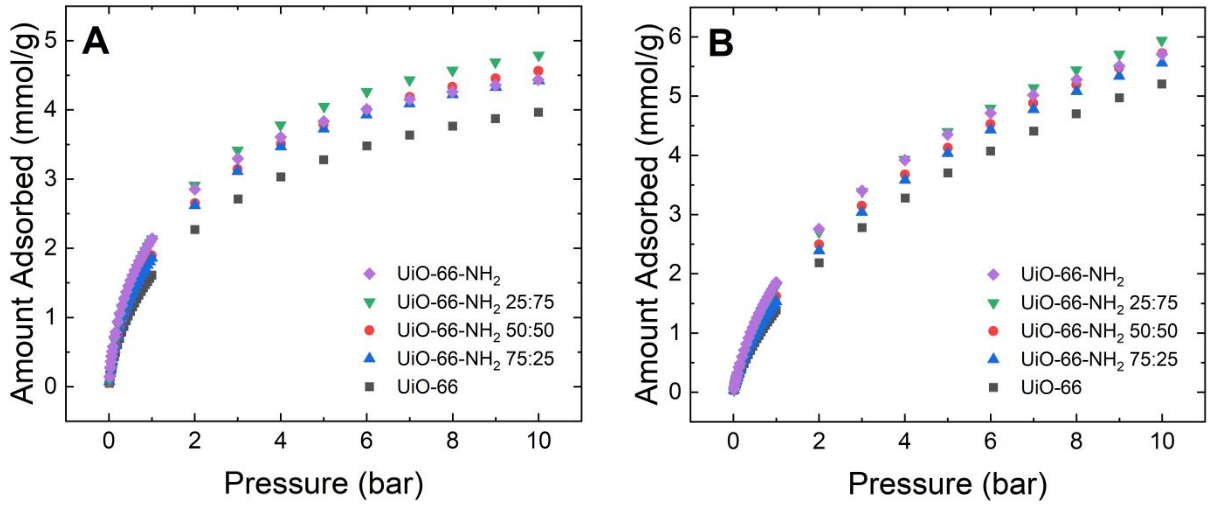

Figure S8. Isotherms from IGA-003 over pressure range up to 10 bar for (A) ethylene and (B) carbon dioxide. Numerical values are tabulated in Table S6-S15.

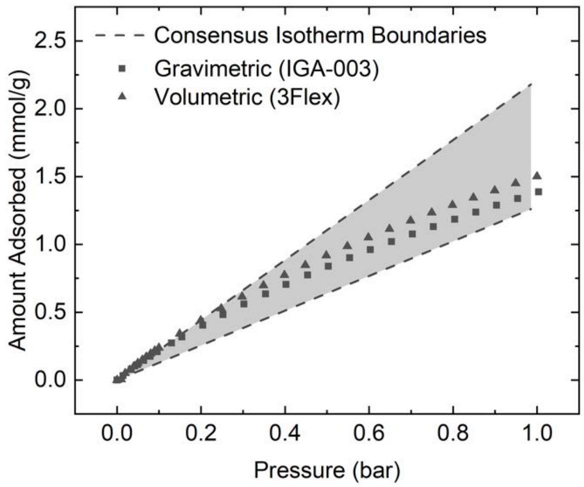

Figure S9. Comparison of the two experimentally measured carbon dioxide isotherms at 298 K (gravimetric and volumetric measurements) to the consensus isotherm for carbon dioxide adsorption in UiO-66 at 298 ± 5 K from literature meta-analysis[3].

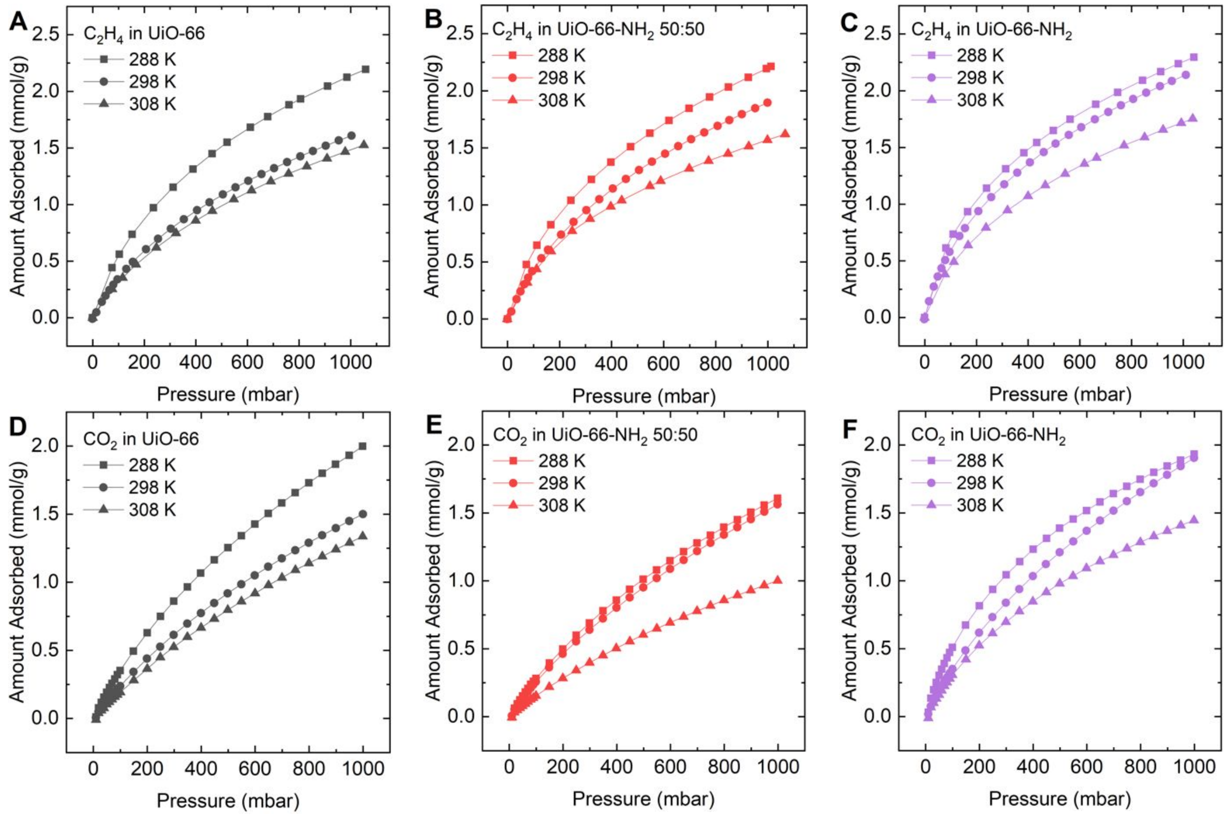

Figure S10. Isotherms measured gravimetrically for ethylene (top) and volumetrically for carbon dioxide (bottom) in (A,D) UiO-66, (B,E) UiO-66-NH_2 50:50, and (C,F) UiO-66-NH_2 at 288, 298, and 308 K. Numerical values are tabulated in Table S17-S31.

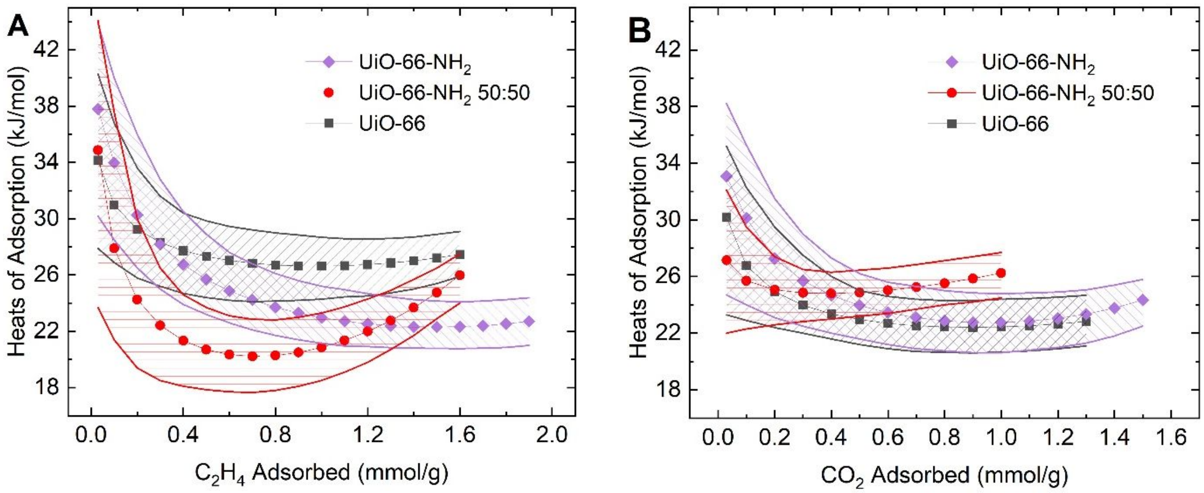

Figure S11. Loading dependent isosteric heats of adsorption for (A) ethylene and (B) carbon dioxide in the pristine UiO-66, the mixed-linker UiO-66-NH_2 50:50, and the fully functionalized UiO-66-NH_2 determined using the Clausius-Clapeyron equation based on isotherms measured at 288, 298, and 308 K. Uncertainty intervals were determined by adding a normally distributed error to each data point in the isotherms, refitting the data, and then calculating maximum and minimum values.

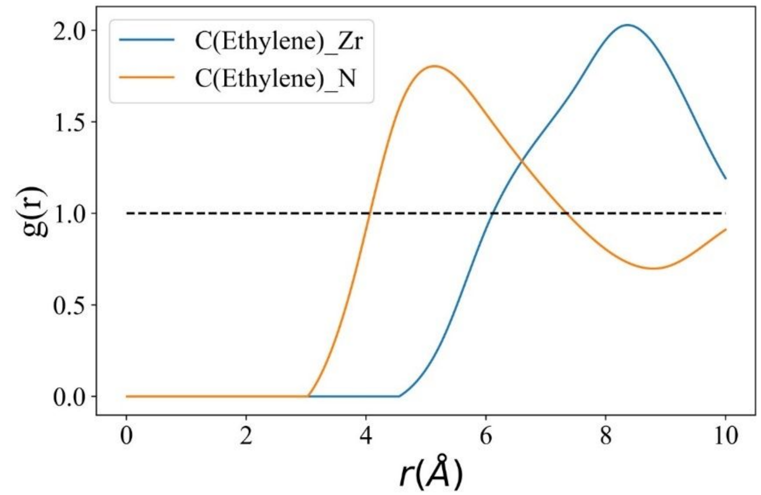

Figure S12. The radial distribution function of the carbon atom in ethylene with respect to Zr and N in the mixed-linker UiO-66-NH_2 50:50. 100,000 configurations are used to calculate the distribution.

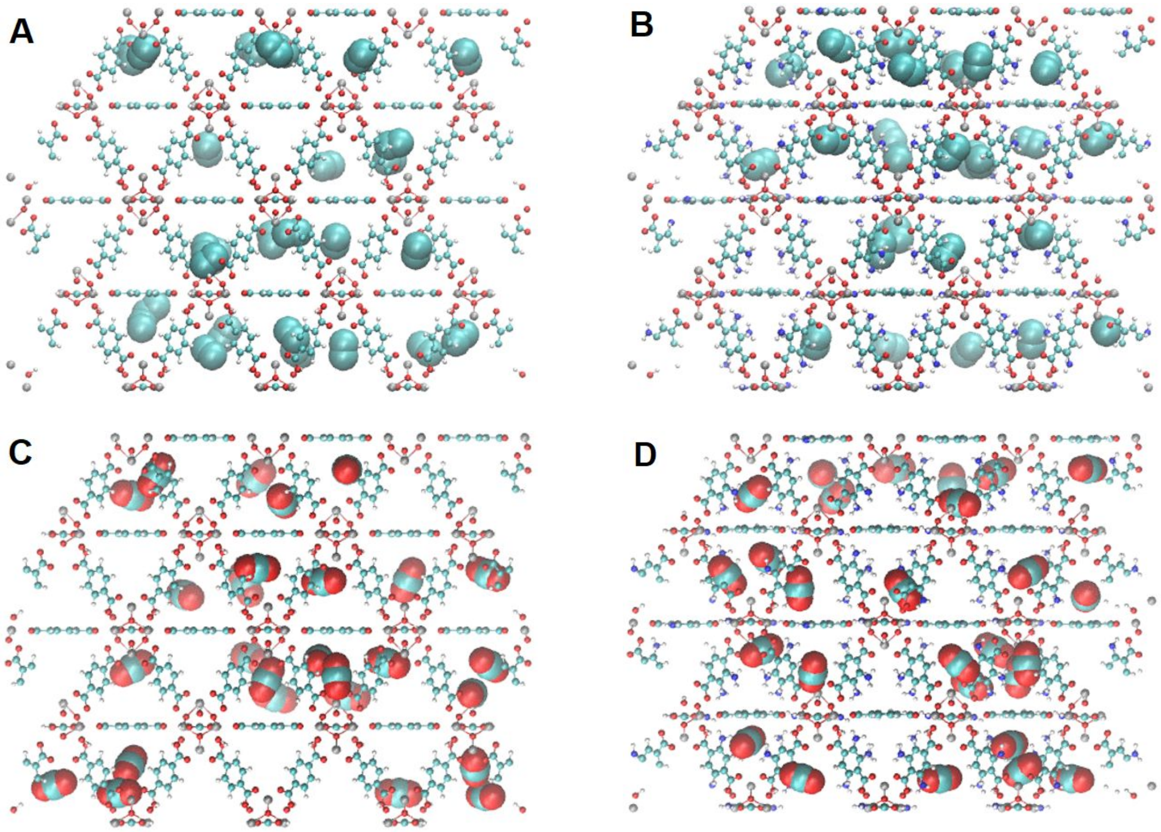

Figure S13. Visualization of a loadings of 0.5 mmol/g of ethylene (top) and carbon dioxide (bottom) in (A,C) UiO-66 and (B,D) UiO-66-NH_2 from GCMC simulations.

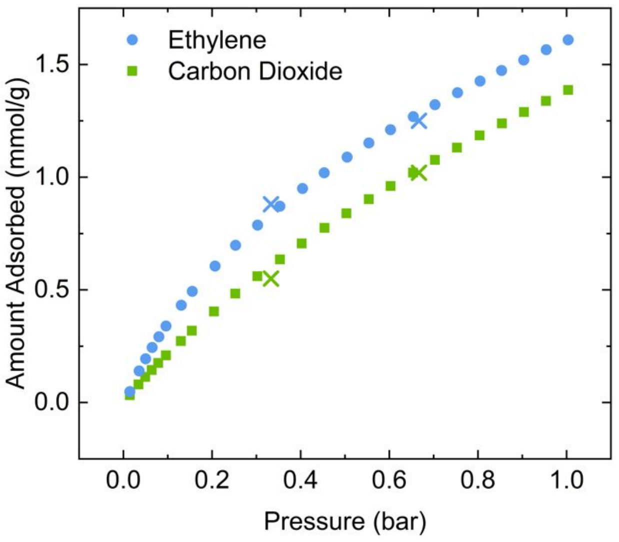

Figure S14. Isotherms for carbon dioxide and ethylene at 298 K (●) compared to dynamic single-component breakthrough capacities (X) in UiO-66.

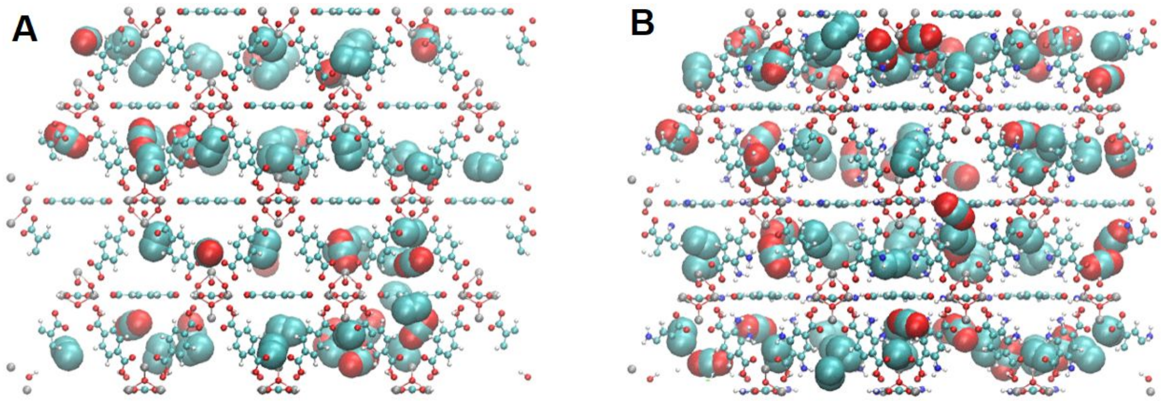

Figure S15. Visualization of the experimentally-determined loadings of ethylene and carbon dioxide using an equimolar mixture at a total, carrier-free pressure of 0.667 bar in (A) UiO-66 and (B) UiO-66-NH_2 from GCMC simulations.

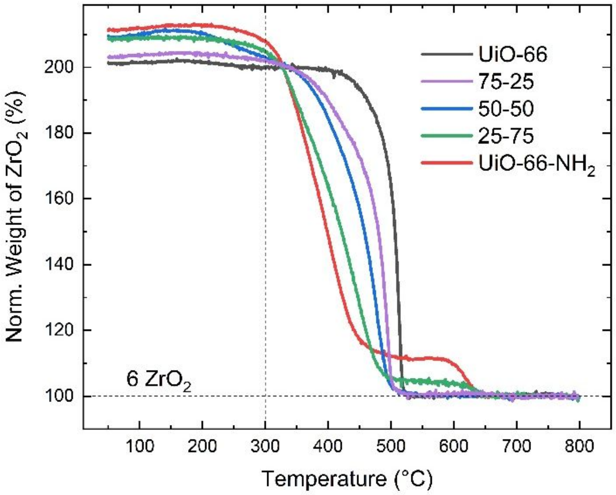

Figure S16. Thermogravimetric analysis curves for five UiO-66-NH_2 mixed-linker derivatives under oxidizing atmosphere from air flow using a heating rate of 2 °C/min. All weights are normalized using the plateau after full decomposition assuming a complete oxidation to zirconium dioxide.

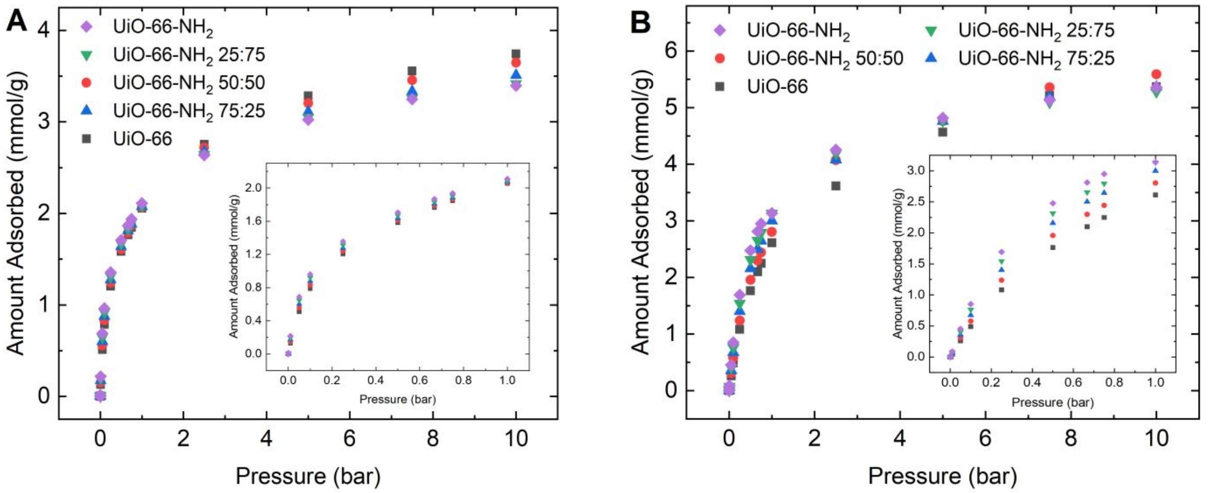

Figure S17. GCMC single-component isotherms for (A) ethylene and (B) carbon dioxide in UiO-66, UiO-66-amine, and the three UiO-66-NH_2 mixed-linker derivatives. Insets show the zoomed part to the low pressure range.

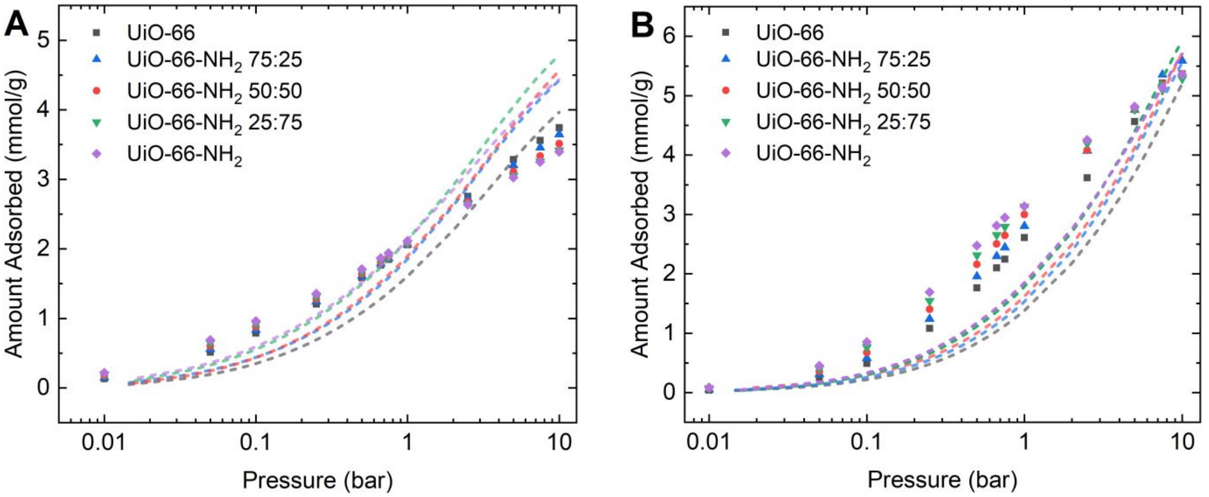

Figure S18. Comparison of GCMC simulated single-component isotherms as symbols to fits of experimentally measured isotherms as dashed lines for (A) ethylene and (B) carbon dioxide at 298 K. The pressure axis is plotted on a logarithmic scale.

S4 Supplementary Tables

Table S1. Suppliers and purities of chemicals and gases used in this study

Chemical/gas	Supplier	Purity [%]
Zirconium(IV)chloride	Sigma Aldrich	99.9
1,4-benzenedicarboxylic acid	Sigma Aldrich	98
2-Aminobenzene-1,4-dicarboxylic acid	Sigma Aldrich	99
N,N-dimethylformamide	Sigma Aldrich	99.8
Methanol	Sigma Aldrich	99.8
Carbon dioxide	Airgas	99.9 (Bone Dry)
Ethylene	Airgas	99.9 (UHP)
Helium	Airgas	99.999 (UHP)
Nitrogen	Airgas	99.999 (UHP)
Air	Airgas	Ultra Zero

Table S2. Lennard-Jones parameters used for the GCMC simulations in this study. The simulation inputs derived based on these parameters are available as Supporting Information usable with RASPA.

Atom	σ [Å]	ε/k_B [K]
Zr	34.7	2.8
C	47.9	3.5
H	7.6	2.8
O	48.2	3.0
N	38.9	3.3
He	10.9	2.6
O_CO_2	79.0	3.1
C_CO_2	27.0	2.8
$CH_2\_sp^2$	85.0	3.7

Table S3. Results of defect quantification based on TGA curves

y_{NMR} (BDC-amine)	M [g/mol]	$W_{theo,Pl}$ [%]	$W_{t,Pl,theo}$ [%]	$W_{ex,Pl}$ [%]	X
0	1628.0	220.2	20.03	199.61	1.03
30	1655.1	223.9	20.64	202.24	1.05
54	1676.7	226.8	21.13	202.86	1.13
75	1695.6	229.3	21.56	204.53	1.15
100	1718.1	232.4	22.06	207.83	1.11

Table S4. Fitting parameter for carbon dioxide adsorption isotherms tabulated in Table S23-S31 and plotted in Figure S10D-F using the Langmuir-Freundlich isotherm model.

CO_2	q_1 [mmol/g]	n_1	k_1 [bar^{-1}]	R^2
UiO-66				
288 K	5.51072	0.921373	0.000536306	0.999948
298 K	4.3137	0.960232	0.000516074	0.999965
308 K	3.99488	0.994838	0.000498224	0.99992
UiO-66-NH_2 50:50				
288 K	4.45944	0.923683	0.000534221	0.999919
298 K	5.67901	0.89538	0.000327107	0.999993
308 K	3.23768	0.957617	0.00043087	0.999932
UiO-66-NH_2				
288 K	3.52198	0.854837	0.00122624	0.999895
298 K	4.99016	0.920672	0.000559294	0.999981
308 K	2.75007	0.951518	0.0010853	0.999822

Table S5. Fitting parameter for ethylene adsorption isotherms tabulated in Table S11, S13, S15, and S17-S22 and plotted in Figure S10A-C using the Langmuir-Freundlich isotherm model.

C_2H_4	q_1 [mmol/g]	n_1	k_1 [bar^{-1}]	R^2
UiO-66				
288 K	4.93683	0.787339	0.000709561	0.999998
298 K	3.49543	0.884785	0.000816748	0.999949
308 K	4.01734	8.35E-01	0.000525271	0.999996
UiO-66-NH_2 50:50				
288 K	5.88539	0.724946	0.000488258	0.999995
298 K	4.51632	0.814577	0.000629642	0.999949
308 K	3.14173	0.823283	0.000982779	0.999893
UiO-66-NH_2				
288 K	5.82704	0.667456	0.000500129	0.999997
298 K	4.48768	0.775102	0.000858178	0.999957
308 K	4.51598	0.741248	0.000518836	0.999995

Table S6. Carbon dioxide in UiO-66 (T = 298 K).

Pressure bar	Uptake mmol/g
0.01469	0.03231
0.03411	0.08004
0.04938	0.11325
0.06398	0.14423
0.07885	0.17498
0.09626	0.20899
0.12988	0.27277
0.15453	0.31818
0.20462	0.40374
0.25271	0.48297
0.30253	0.56024
0.35357	0.63484
0.40246	0.70542
0.45403	0.77461
0.50332	0.83890
0.55395	0.90224
0.60311	0.96039
0.65387	1.01961
0.7033	1.07611
0.75299	1.13059
0.80389	1.18508
0.85439	1.23835
0.90395	1.28855
0.95378	1.33782
1.0036	1.38633
2.00551	2.1818
3.00474	2.77933
4.00759	3.27576
5.00735	3.69998
6.00337	4.07027
7.00514	4.40557
7.99902	4.70101
9.00307	4.96916
9.99466	5.20509

Table S7. Carbon dioxide in UiO-66-NH_2 75:25 (T = 298 K).

Pressure bar	Uptake mmol/g
0.01469	0.04063
0.03371	0.09708
0.0475	0.13252
0.06344	0.17061
0.07778	0.20508
0.09425	0.24227
0.13149	0.32199
0.15332	0.36771
0.20636	0.46879
0.25606	0.55775
0.30495	0.64084
0.35317	0.71846
0.40286	0.79489
0.45309	0.86927
0.50479	0.94185
0.55515	1.01076
0.60204	1.07207
0.65293	1.13688
0.70142	1.19665
0.75473	1.26054
0.80443	1.31818
0.85372	1.37409
0.90234	1.42781
0.95324	1.48199
1.0036	1.53544
2.00082	2.38864
2.99724	3.04136
4.00317	3.58395
4.99958	4.03577
5.99841	4.43045
6.99965	4.77355
8.00544	5.07851
8.99918	5.33649
9.99774	5.55908

Table S8. Carbon dioxide in UiO-66-NH_2 50:50 (T = 298 K).

Pressure bar	Uptake mmol/g
0.01924	0.08185
0.03706	0.14011
0.05099	0.17647
0.06492	0.21085
0.07992	0.24864
0.09599	0.28536
0.13068	0.36053
0.15466	0.41227
0.20395	0.50786
0.25311	0.60220
0.30414	0.69492
0.35357	0.77765
0.40286	0.85698
0.45336	0.93602
0.50305	1.00874
0.55261	1.07850
0.60204	1.14637
0.65307	1.21233
0.70316	1.27614
0.75219	1.33642
0.80242	1.39764
0.85238	1.45624
0.90154	1.51171
0.95203	1.56769
1.00186	1.62208
2.00109	2.49583
2.99818	3.14685
4.0037	3.67659
5.00106	4.12756
5.99694	4.52726
6.99295	4.87899
8.00076	5.19423
8.99423	5.47524
10.00029	5.72578

Table S9. Carbon dioxide in UiO-66-NH_2 25:75 (T = 298 K).

Pressure bar	Uptake mmol/g
0.01616	0.03665
0.03679	0.11148
0.05032	0.15583
0.06532	0.20153
0.07938	0.24381
0.09599	0.29033
0.13376	0.38922
0.15653	0.44746
0.20864	0.56715
0.25659	0.67014
0.30575	0.77017
0.35611	0.86709
0.40554	0.95658
0.45389	1.03900
0.50506	1.12348
0.55355	1.19907
0.60338	1.27191
0.65481	1.34615
0.7029	1.41490
0.75326	1.48318
0.80349	1.54565
0.85291	1.61169
0.9018	1.67122
0.95351	1.73041
1.0024	1.78952
1.99801	2.70401
3.00193	3.38998
3.99205	3.93131
4.99865	4.40066
5.99627	4.79503
7.00313	5.13948
7.99768	5.44126
9.00146	5.70752
9.9893	5.93899

Table S10. Carbon dioxide in UiO-66-NH_2 (T = 298 K).

Pressure bar	Uptake mmol/g
0.01589	0.04956
0.03639	0.12861
0.05206	0.18377
0.06599	0.23003
0.08032	0.27507
0.09693	0.32470
0.13109	0.41700
0.15533	0.48199
0.20462	0.59809
0.25405	0.70943
0.30361	0.81770
0.35477	0.91693
0.4034	1.00988
0.45376	1.09757
0.50426	1.18187
0.55435	1.26310
0.60338	1.33721
0.65521	1.41343
0.70424	1.48181
0.75326	1.54860
0.80456	1.61630
0.85466	1.67925
0.90234	1.73763
0.95445	1.79948
1.00226	1.85499
1.9956	2.75565
3.0022	3.40192
4.00156	3.91796
4.99583	4.35023
5.99922	4.71218
6.99095	5.01808
8.00705	5.28168
8.99771	5.50874
9.99252	5.70640

Table S11. Ethylene in UiO-66 (T = 298 K)

Pressure bar	Uptake mmol/g
0.01455	0.04860
0.03558	0.14023
0.05018	0.19391
0.06505	0.24409
0.08005	0.29162
0.09639	0.33932
0.13068	0.43143
0.1556	0.49334
0.20676	0.60536
0.25311	0.69754
0.3028	0.78682
0.3533	0.87068
0.40433	0.94963
0.45309	1.01911
0.50479	1.08862
0.55395	1.15149
0.60297	1.21025
0.65427	1.26840
0.7033	1.32123
0.75406	1.37487
0.80496	1.42622
0.85358	1.47313
0.90341	1.51963
0.95418	1.56569
1.00374	1.60894
2.00257	2.26952
3.00715	2.71137
4.00236	3.03063
5.0024	3.27828
6.0035	3.47788
6.99992	3.63312
8.00143	3.76278
8.99972	3.87367
9.99895	3.96530

Table S12. Ethylene in UiO-66-NH_2 75:25 (T = 298 K).

Pressure bar	Uptake mmol/g
0.01455	0.07483
0.03692	0.19480
0.05099	0.25895
0.06425	0.31141
0.07965	0.36972
0.09639	0.42665
0.13109	0.53183
0.15479	0.59915
0.20475	0.72092
0.25351	0.82884
0.30495	0.93301
0.35424	1.02325
0.40353	1.10813
0.45322	1.18805
0.50399	1.26384
0.55315	1.33600
0.60364	1.40444
0.65401	1.46857
0.7037	1.53006
0.75339	1.58898
0.80416	1.64640
0.85452	1.70329
0.90301	1.75514
0.95378	1.80694
1.00454	1.85809
2.00176	2.61867
2.99496	3.11224
4.00464	3.46679
5.00133	3.72733
5.99654	3.92878
6.99764	4.08911
8.00049	4.22052
9.00668	4.32807
10.00658	4.42114

Table S13. Ethylene in UiO-66-NH_2 50:50 (T = 298 K).

Pressure bar	Uptake mmol/g
0.01536	0.06610
0.03505	0.17378
0.04991	0.24113
0.06465	0.30351
0.07952	0.36175
0.09532	0.41846
0.13068	0.53281
0.1556	0.60657
0.20609	0.73908
0.25431	0.85125
0.3028	0.95355
0.35303	1.04919
0.40473	1.14335
0.45537	1.22661
0.50546	1.30505
0.55502	1.37876
0.60565	1.44770
0.65575	1.51367
0.70544	1.57570
0.75594	1.63470
0.80737	1.69248
0.85225	1.74281
0.90073	1.79541
0.9515	1.84671
0.99891	1.89611
2.00752	2.65041
2.99697	3.14311
4.00491	3.50873
4.9953	3.78584
6.00404	4.00739
7.00421	4.18638
7.99473	4.33150
8.99757	4.45389
9.99439	4.56353

Table S14. Ethylene in UiO-66-NH_2 25:75 (T = 298 K).

Pressure bar	Uptake mmol/g
0.01603	0.10523
0.03598	0.24803
0.05072	0.32980
0.06532	0.40614
0.07858	0.46660
0.09653	0.53978
0.13229	0.67044
0.15587	0.74790
0.20650	0.89334
0.25338	1.01171
0.30508	1.13090
0.35504	1.23386
0.40353	1.32667
0.45282	1.41456
0.50412	1.49833
0.55315	1.57542
0.60338	1.64744
0.65468	1.71938
0.7045	1.78494
0.75406	1.84889
0.80456	1.91004
0.85278	1.96742
0.90489	2.02701
0.95431	2.08014
1.00441	2.13384
1.99721	2.91199
2.99751	3.41685
3.9962	3.77927
5.00682	4.04939
6.0027	4.26279
6.99644	4.43359
7.99406	4.57320
8.99436	4.69020
10.00189	4.78762

Table S15. Ethylene in UiO-66-NH_2 (T = 298 K).

Pressure bar	Uptake mmol/g
0.01670	0.14342
0.03531	0.27221
0.05045	0.36160
0.06451	0.43471
0.07952	0.50617
0.09639	0.57842
0.1343	0.71913
0.15546	0.78870
0.20757	0.93828
0.25713	1.06185
0.30736	1.17391
0.35799	1.27678
0.40728	1.36811
0.46032	1.45911
0.50653	1.53399
0.5573	1.61009
0.60552	1.67752
0.65856	1.74779
0.71013	1.81268
0.75902	1.87118
0.8067	1.92624
0.8572	1.98223
0.91065	2.03943
0.95632	2.08571
1.01057	2.13949
2.00297	2.85249
3.00153	3.29565
4.00102	3.60710
5.00267	3.83441
5.99988	4.01062
6.99952	4.15143
7.99607	4.26246
8.99865	4.35868
9.99131	4.43481

Table S16. Fitting parameter for isotherms listen in Table S6-S15 used for IAST calculations.

	q_1	n_1	k_1	q_2	n_2	k_2	R^2
Carbon Dioxide							
UiO-66	4.66488	0.930901	0.277559	0.611539	1.09821	4.77752	0.999998
75:25	0.499802	1.11625	8.4176	5.14699	0.936697	0.349193	0.999998
50:50	0.692839	1.1236	5.46174	5.37198	0.913522	0.283593	0.999997
25:75	0.499738	1.21932	9.99466	5.7669	0.858019	0.347327	0.999997
UiO-66-NH_2	4.7384	0.893514	0.391226	0.794358	0.985512	7.62566	0.999999
Ethylene							
UiO-66	1.53589	0.951182	0.908333	9.20948	0.974938	0.070941	0.999999
75:25	2.34277	0.906912	0.829636	6.7013	1.18117	0.109212	0.999999
50:50	8.71521	0.812538	0.162189	0.803965	3.14405	0.121102	0.999999
25:75	8.74895	0.991567	0.114416	1.33532	1.03645	1.81105	0.999998
UiO-66-NH_2	1.71382	0.967654	1.50957	7.43434	0.998685	0.124145	0.999997

Table S17. Ethylene in UiO-66 (T = 288 K).

Pressure mbar	Uptake mmol/g
74.916	0.4423
103.382	0.56058
153.231	0.73685
235.69	0.97120
311.733	1.15330
388.979	1.31306
463.152	1.44967
521.153	1.54993
611.096	1.68253
678.319	1.77568
760.777	1.88029
805.949	1.93329
910.458	2.04670
985.433	2.12414
1058.001	2.19430

Table S18. Ethylene in UiO-66 (T = 308 K).

Pressure mbar	Uptake mmol/g
75.509	0.25277
116.136	0.35356
168.124	0.47016
246.84	0.62095
323.017	0.74725
399.595	0.85862
463.611	0.94416
547.005	1.04682
615.698	1.12442
690.538	1.20430
759.365	1.27248
829.929	1.33832
908.111	1.40668
979.076	1.46670
1050.976	1.52469

Table S19. Ethylene in UiO-66-NH_2 50:50 (T = 288 K).

Pressure mbar	Uptake mmol/g
73.161	0.47676
113.655	0.64697
166.578	0.82610
243.824	1.04054
322.273	1.22278
397.782	1.37357
473.157	1.50915
546.929	1.62897
620.166	1.73908
697.546	1.84613
776.529	1.94518
849.232	2.03242
924.874	2.11825
994.77	2.19389
1012.678	2.21382

Table S20. Ethylene in UiO-66-NH_2 50:50 (T = 308 K

Pressure mbar	Uptake mmol/g
76.602	0.31933
112.151	0.43770
167.213	0.59481
248.735	0.77162
317.295	0.87832
396.679	0.98603
439.045	1.03989
547.43	1.16576
588.592	1.21080
698.18	1.31814
772.62	1.38623
847.193	1.44860
926.712	1.51322
998.746	1.56948
1066.236	1.61939

Table S21. Ethylene in UiO-66-NH_2 (T = 288 K).

Pressure mbar	Uptake mmol/g
81.6140	0.61349
110.080	0.73494
165.676	0.93270
239.448	1.14059
313.353	1.31077
383.516	1.45263
433.499	1.54371
497.648	1.64968
562.465	1.74884
661.362	1.88113
746.493	1.98574
842.984	2.09101
913.014	2.16756
981.038	2.23683
1041.312	2.29581

Table S22. Ethylene in UiO-66-NH_2 (T = 308 K).

Pressure mbar	Uptake mmol/g
78.5060	0.38122
112.852	0.49196
167.646	0.63798
237.007	0.79262
320.000	0.94528
399.385	1.07110
466.474	1.16701
543.32	1.26790
617.626	1.35627
665.069	1.40962
771.049	1.51913
850.701	1.58951
922.067	1.65623
992.096	1.71528
1036.60	1.75313

Table S23. Carbon dioxide in UiO-66 (T = 288 K).

Pressure mbar	Uptake mmol/g
10.0181	0.01051
19.4023	0.07822
30.1813	0.11852
39.6085	0.15559
49.9353	0.19147
60.3976	0.22634
69.7166	0.25740
79.7231	0.28986
89.7339	0.32105
99.709	0.35213
148.76	0.49405
199.758	0.62897
249.104	0.74868
298.662	0.86090
348.76	0.96598
397.904	1.06726
448.779	1.16490
499.008	1.25494
548.915	1.34149
599.032	1.42643
648.701	1.50568
698.874	1.58225
748.402	1.65746
799.11	1.73038
849.188	1.79967
898.229	1.86679
949.094	1.93257
998.718	1.99864

Table S24. Carbon dioxide in UiO-66 (T = 298 K).

Pressure mbar	Uptake mmol/g
10.0294	0.00541
19.4874	0.05027
30.1674	0.07537
39.7312	0.09962
49.6747	0.12571
60.1952	0.14952
69.6833	0.17201
79.9845	0.19530
90.0000	0.21700
99.7030	0.23853
149.247	0.34196
199.269	0.43919
248.011	0.52729
297.894	0.61413
348.583	0.69706
398.835	0.77369
448.158	0.84776
498.327	0.91893
548.461	0.98573
598.555	1.05073
648.704	1.11360
698.972	1.17516
749.192	1.23420
798.750	1.28993
848.658	1.34451
899.078	1.39756
948.956	1.44972
999.951	1.50082

Table S25. Carbon dioxide in UiO-66 (T = 308 K).

Pressure mbar	Uptake mmol/g
10.1860	-0.01005
19.8962	0.03931
30.1590	0.05905
39.7579	0.07668
49.8575	0.10413
59.5894	0.12140
69.7034	0.14003
80.1286	0.15873
89.7240	0.17453
100.204	0.19244
150.210	0.28045
199.865	0.36470
248.688	0.44844
298.083	0.52414
348.612	0.59884
399.777	0.66723
448.562	0.73165
498.586	0.79668
549.371	0.85904
599.457	0.91927
649.549	0.97909
699.661	1.03446
749.563	1.09034
799.179	1.13946
849.104	1.19019
899.459	1.24115
950.213	1.29082
998.639	1.33687

Table S26. Carbon dioxide in UiO-66-NH_2 50:50 (T = 288 K).

Pressure mbar	Uptake mmol/g
10.1344	-0.00293
19.6039	0.06335
30.1373	0.09457
39.7272	0.12345
49.8759	0.15345
59.8471	0.18174
69.6781	0.20784
80.0641	0.23851
89.8772	0.26014
99.6313	0.28201
149.339	0.39548
199.356	0.49892
248.971	0.59954
298.330	0.69017
348.858	0.77929
398.913	0.85832
448.020	0.93746
498.641	1.01211
548.543	1.07977
598.067	1.14878
648.691	1.21541
698.431	1.27839
748.762	1.33525
798.366	1.39445
848.872	1.45073
898.645	1.50486
948.927	1.55801
998.801	1.60795

Table S27. Carbon dioxide in UiO-66-NH_2 50:50 (T = 298 K).

Pressure mbar	Uptake mmol/g
10.0139	0.00732
19.4408	0.05431
30.1505	0.08163
39.7003	0.11348
50.1312	0.13963
60.0170	0.16362
69.7802	0.18734
79.9661	0.21199
89.7873	0.23450
99.6077	0.25707
148.658	0.36200
199.276	0.46269
249.211	0.55477
298.901	0.64039
348.692	0.72280
398.853	0.80172
448.149	0.87722
498.986	0.95166
549.071	1.02038
598.911	1.08818
648.917	1.15329
698.868	1.21831
748.955	1.27979
799.010	1.33854
848.537	1.39472
898.936	1.45314
949.431	1.50849
998.621	1.56280

Table S28. Carbon dioxide in UiO-66-NH_2 50:50 (T = 308 K).

Pressure mbar	Uptake mmol/g
10.1491	-0.00445
19.6071	0.03307
30.3060	0.04909
39.9794	0.06456
50.0191	0.07896
59.7668	0.09543
69.7159	0.11026
79.7831	0.12662
89.6431	0.13906
99.5192	0.15385
148.864	0.22035
199.097	0.28316
248.857	0.34218
298.970	0.39866
348.848	0.45224
398.369	0.50456
449.022	0.55447
498.827	0.60394
549.135	0.64908
598.544	0.69213
648.930	0.73675
698.993	0.77790
748.936	0.81778
799.017	0.85768
849.171	0.89412
899.221	0.93056
948.522	0.96722
998.916	1.00235

Table S29. Carbon dioxide in UiO-66-NH_2 (T = 288 K).

Pressure mbar	Uptake mmol/g
9.91306	0.03257
19.5378	0.13541
30.0819	0.19800
39.7686	0.25169
50.1390	0.30451
60.1133	0.34908
69.7400	0.39158
80.0683	0.43335
89.7265	0.47243
99.7475	0.51041
149.092	0.67329
199.999	0.81608
249.710	0.93701
299.780	1.04334
350.193	1.14169
400.317	1.23312
450.230	1.31254
498.727	1.38755
548.847	1.45520
598.728	1.51747
648.748	1.58140
698.814	1.64151
748.671	1.69545
798.640	1.74821
848.794	1.79771
898.989	1.84428
948.775	1.88827
999.032	1.93334

Table S30. Carbon dioxide in UiO-66-NH_2 (T = 298 K).

Pressure mbar	Uptake mmol/g
9.90260	0.01896
19.3622	0.07684
30.1078	0.11738
39.6864	0.15530
49.9312	0.19081
59.8009	0.22472
70.1789	0.25968
79.9832	0.29064
89.7388	0.32103
99.7224	0.35139
148.293	0.48803
199.327	0.61760
249.118	0.73249
298.893	0.83927
348.795	0.93905
398.898	1.03382
447.910	1.12331
499.093	1.20950
549.193	1.29031
599.091	1.36845
649.027	1.44353
698.996	1.51561
749.035	1.58568
799.143	1.65284
848.620	1.71767
898.385	1.78084
949.375	1.84450
998.689	1.90463

Table S31. Carbon dioxide in UiO-66-NH_2 (T = 308 K).

Pressure mbar	Uptake mmol/g
10.1356	-0.01068
19.7897	0.06852
30.1650	0.10026
39.9098	0.13205
50.1467	0.16097
59.7092	0.19254
70.3522	0.22514
80.0530	0.25127
90.2524	0.27967
99.9665	0.30649
150.021	0.42200
199.226	0.52380
249.395	0.61421
298.865	0.69742
349.043	0.77552
398.984	0.84836
449.092	0.91550
499.140	0.97936
548.213	1.03503
598.531	1.09294
648.906	1.14339
698.992	1.19042
749.009	1.23840
798.967	1.28425
849.040	1.32800
899.207	1.36765
949.453	1.40817
998.584	1.44577

S5 Supplementary References

1. Shearer, G. C.; Chavan, S.; Bordiga, S.; Svelle, S.; Olsbye, U.; Lillerud, K. P., Defect Engineering: Tuning the Porosity and Composition of the Metal–Organic Framework UiO-66 via Modulated Synthesis. *Chem. Mater.* **2016,** *28*, 3749-3761.

2. Sannes, D. K.; Øien-Ødegaard, S.; Aunan, E.; Nova, A.; Olsbye, U., Quantification of Linker Defects in UiO-Type Metal–Organic Frameworks. *Chem. Mater.* **2023,** *35*, 3793-3800.

3. Park, J.; Howe, J. D.; Sholl, D. S., How Reproducible Are Isotherm Measurements in Metal–Organic Frameworks? *Chem. Mater.* **2017,** *29*, 10487-10495.

4. Bingel, L. W.; Chen, A.; Agrawal, M.; Sholl, D. S., Experimentally Verified Alcohol Adsorption Isotherms in Nanoporous Materials from Literature Meta-Analysis. *J. Chem. Eng. Data* **2020,** *65*, 4970-4979.
