# Supplementary figures and images for: Does Mixed Linker-Induced Surface Heterogeneity Impact the Accuracy of IAST Predictions in UiO-66-NH2?
Source: J Phys Chem C Nanomater Interfaces. 2023 Oct 12;127(42):20881–9. doi: 10.1021/acs.jpcc.3c04845 (PMC10614300; doi:10.1021/acs.jpcc.3c04845)

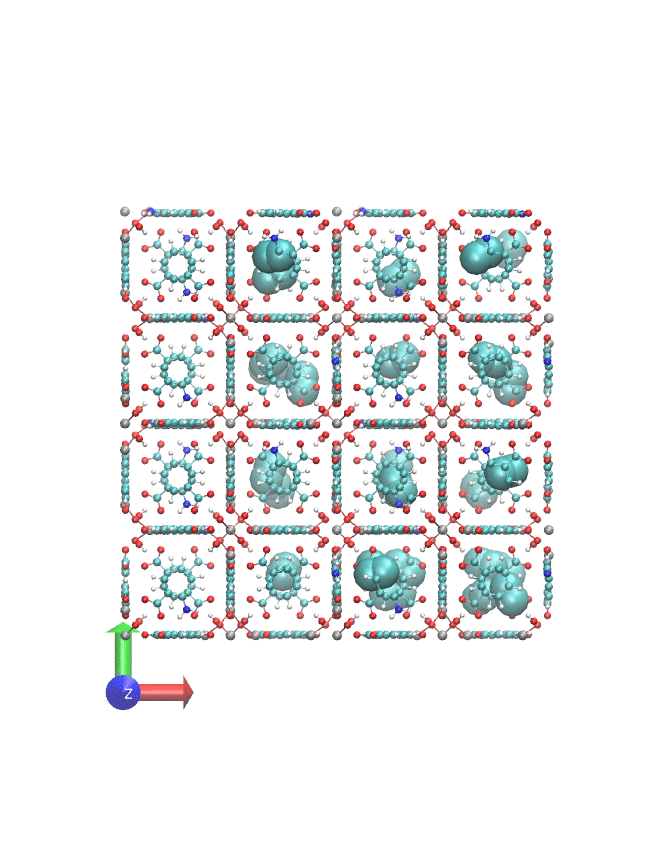

Supplement: Supplementary file 4 — jp3c04845_si_004.zip [file jp3c04845_si_004.zip › GIFs MC cycles/C2H4_0.5_UiO66-Mix.gif]
